# Interventions to Support Mental Health among Those with Health Conditions That Present Risk for Severe Infection from Coronavirus Disease 2019 (COVID-19): A Scoping Review of English and Chinese-Language Literature †

**DOI:** 10.3390/ijerph18147265

**Published:** 2021-07-07

**Authors:** Karen M. Davison, Vidhi Thakkar, Shen (Lamson) Lin, Lorna Stabler, Maura MacPhee, Simon Carroll, Benjamin Collins, Zachary Rezler, Jake Colautti, Chaoqun (Cherry) Xu, Esme Fuller-Thomson, Brandon Hey, Krystal Kelly, Laura Mullaly, Ron Remick, Arun Ravindran, Angela Paric, Carla D’Andreamatteo, Victoria Smye

**Affiliations:** 1 Health Science Program, Kwantlen Polytechnic University, 12666 72 Ave, Surrey, BC V3W 2M8, Canada; vidhi.thakkar@kpu.ca; 2 Factor-Inwentash Faculty of Social Work, University of Toronto, 46 Bloor St W, Toronto, ON M5S 1V4, Canada; lamsonlin.lin@mail.utoronto.ca (S.L.); esme.fuller.thomson@utoronto.ca (E.F.-T.); 3 CASCADE Children’s Social Care Research and Development Centre, School of Social Sciences, Cardiff University, 1-3 Museum Place, Cardiff CF10 3BD, UK; StablerL@Cardiff.ac.uk; 4 School of Nursing, University of British Columbia, T201-2211 Wesbrook Mall, Vancouver, BC V6T 2B5, Canada; Maura.MacPhee@ubc.ca; 5 Department of Sociology, Cornett Building, University of Victoria, A333, Victoria, BC V8W 3P5, Canada; scarroll@uvic.ca; 6 Department of Anthropology, University of Manitoba, 432 Fletcher Argue Building, 15 Chancellor Circle, Winnipeg, MB R3T 2N2, Canada; Benjamin.Collins@umanitoba.ca; 7 Health Sciences Program, McMaster University, 1280 Main Street West, Hamilton, ON L8S 4K1, Canada; rezlerz@mcmaster.ca (Z.R.); colauttj@mcmaster.ca (J.C.); xuc73@mcmaster.ca (C.X.); 8 COVID 19 Policy, Programs and Priorities, Mental Health Commission of Canada, 350 Albert Street, Suite 1210, Ottawa, ON K1R 1A4, Canada; bhey@mentalhealthcommission.ca; 9 Mental Health Advancement, Mental Health Commission of Canada, 350 Albert Street, Suite 1210, Ottawa, ON K1R 1A4, Canada; lmullaly@mentalhealthcommission.ca (L.M.); kkelly@mentalhealthcommission.ca (K.K.); 10 Lookout Housing and Health Society, 544 Columbia St, New Westminster, BC V3L 1B1, Canada; ron.remick@lookoutsociety.ca; 11 Campbell Family Mental Health Research Institute, The Centre for Addiction and Mental Health, 1001 Queen St W, Toronto, ON M6J 1H4, Canada; Arun.Ravindran@camh.ca (A.R.); Angela.Paric@camh.ca (A.P.); 12 Department of Food and Human Nutritional Sciences, University of Manitoba, 209 Human Ecology Building, Winnipeg, MB R3T 2N2, Canada; carla@thefoodlady.ca; 13 Arthur Labatt School of Nursing, Western University, 1151 Richmond Street, London, ON N6A 3K7, Canada; vsmye@uwo.ca

**Keywords:** COVID-19, mental health, substance use, chronic diseases

## Abstract

This study aimed to address knowledge gaps related to the prevention and management of mental health responses among those with a condition that presents risk of severe COVID-19 infection. A scoping review that mapped English and Chinese-language studies (2019–2020) located in MEDLINE (Ovid), Cumulative Index to Nursing and Allied Health Literature (CINAHL), PsycInfo, Sociological Abstracts, Embase, China National Knowledge Infrastructure (CNKI), Wanfang Data, and Airiti Library was undertaken. Search terms related to COVID-19, mental health, and physical health were used and articles that included all three of these factors were extracted (*n* = 77). With the exception of one hospital-based pilot study, there were no intervention studies targeting mental health in those at risk of severe COVID-19 infection. Promising practices such as integrated care models that appropriately screen for mental health issues, address health determinants, and include use of digital resources were highlighted. Patient navigator programs, group online medical visits, peer support, and social prescribing may also support those with complex needs. Future policies need to address digital health access inequities and the implementation of multi-integrated health and social care. Furthermore, research is needed to comprehensively assess multi-integrated interventions that are resilient to public health crises.

## 1. Introduction

The COVID-19 pandemic has presented many mental health challenges, particularly for those with physical health conditions who are at risk for severe novel coronavirus pneumonia [1]. Circumstances which have contributed to poor mental health include the impacts of quarantine (e.g., social isolation), social distancing, altered health care access, being in an extended and uncertain emergency state, unexpected unemployment, economic despair, complicated grief, and fear of morbidity and mortality from COVID-19 infection [2,3,4,5,6]. In accordance with the Mental Health Continuum Model, all people, regardless of pre-existing conditions or diagnoses, have the potential to live a flourishing, joyful life [7]. Although COVID-19 has challenged people’s health and well-being, there is opportunity to better understand how future policies, programs, and initiatives may be modified to strengthen the mental health assets of different populations.

Although there has been much written about generic ways to promote mental health during COVID-19, there is limited information about what specific mental health promotion interventions are most effective for those with physical health conditions that put them at risk of severe infection from contracting COVID-19. Previous literature about interventions to reduce the psychological impact of pandemics is limited. The Economic and Social Research Institute in Dublin produced a working paper [8] which considered pro-social behaviors, communication, risk perception, and the impacts of isolation on health behaviors. A survey conducted during the H1N1 influenza pandemic indicated the importance of precise information for reducing anxiety [9]. A rapid review on the impact of quarantine reported that when compared to mandated approaches, voluntary quarantine contributes to less distress and long-term complications [10].

It is well known that there are many shared determinants such as socioeconomic factors, health behaviors, psychological factors, and environmental factors that contribute to non-communicable and communicable diseases [1,11,12]. However, there are knowledge gaps about these relationships and how they differ across individuals with different health conditions. A better understanding of specific risk factors and mental health supports for individuals with different pre-existing medical conditions during the COVID-19 pandemic will contribute to more effective program and policy interventions [13,14]. To address the needs of various knowledge users (e.g., policy-makers, program planners, health organizations and providers, end users), a scoping review was conducted, based on the following questions:

i. What mental health conditions and substance use risk factors are related to the COVID-19 pandemic among populations with chronic physical health conditions who are at risk of contracting COVID-19 and having severe symptoms?
ii. What are effective health promotion, primary prevention, screening, and treatment interventions to enhance mental health outcomes and to reduce risk of substance use for populations with chronic physical health conditions who are at risk of contracting COVID-19 and having severe symptoms?

## 2. Materials and Methods

### 2.1. Search Strategy

Our scoping review was guided by the Arksey and O’Malley methodology [15]. The literature search aimed to locate English and Chinese-language studies from December 2019 to October 2020. December 2019 was when the Wuhan Municipal Health Commission in China reported the first cluster of cases with severe acute respiratory syndrome coronavirus 2 (SARS-CoV-2) in Wuhan, Hubei Province. The search strategy for English-language literature included the following databases: MEDLINE (Ovid), Cumulative Index to Nursing and Allied Health Literature (CINAHL), PsycInfo, Sociological Abstracts, and Embase. Other English-language search strategies included gray literature sources (e.g., COVID-related bulletins and correspondence) published between December 2019 and October 2020, reference checking, and communications with knowledge users. The Chinese-language studies were searched using three bibliographic databases: two of the largest databases from Mainland China: (1) China National Knowledge Infrastructure (CNKI); (2) Wanfang Data; and one database from Taiwan: (3) Airiti Library. Search terms used were related to COVID-19, mental health, and physical health. Full details about the search strategy are outlined in Supplementary Table S1.

### 2.2. Inclusion/Exclusion Criteria and Search Dates

For both the English and Chinese-language literature searches, the following criteria were applied. Inclusion criteria: (i) English and Chinese-language articles from any country; (ii) full text available; (iii) adults age 18 years+; (iv) COVID-19 content. All study types or methodological approaches were included (i.e., qualitative, quantitative). At least one of the following physical health conditions needed to be included: obesity; diabetes; cancer or related terms (e.g., tumor, neoplasm, malignancy); cardiovascular disease; respiratory disease; autoimmune conditions; kidney disease; hepatitis; HIV or AIDS; frailty; neurocognitive conditions, and/or functional limitations. Finally, eligible studies needed to include at least one of the following mental health conditions: depression; anxiety; bipolar disorder, mania; schizophrenia, schizoaffective; psychosis; obsessive disorders, neurosis; post-traumatic stress disorder (PTSD) or trauma; stress; substance use or related terms (e.g., addiction, compulsive drug abuse, drug dependence); behavioral addiction (e.g., gambling); impulsivity and/or disruptive, and impulse-control and conduct disorders. Exclusion criteria: (i) studies that focused on health care workers; (ii) studies that focused on pregnancy, pediatrics, and populations less than 18 years old; (iii) studies that did not include COVID-19 or the health conditions outlined in the inclusion criteria. The English and Chinese-language literature searches were done at two time points: between 11 and 13 June 2020 with updates between 11 and 17 October 2020.

### 2.3. Screening, Data Extraction, and Quality Assessment

The files from all database searches were imported to Covidence [16]. English literature abstracts and full texts were screened by two reviewers. For the Chinese-language literature, abstracts and full texts were screened by two reviewers who could read and speak Chinese fluently. When there was disagreement regarding the inclusion/exclusion between two reviewers, a senior researcher (K.D., S.L., or V.T.) made the final decision.

Several recognized tools were used to assess the quality of evidence, sort the research studies, and extract the required data. For cohort studies, the Strengthening the Reporting of Observational Studies in Epidemiology (STROBE) checklist was used [17]. For RCTs, systematic reviews, and meta-analyses, the Meta-analyses Of Observational Studies in Epidemiology (MOOSE) checklist was applied [18]. In addition, the Mixed Methods Appraisal Tool (MMAT) was used [19]. After quality assessment, data extraction was conducted where relevant information from each article was entered into a spreadsheet based on the PICOTS (Population, Intervention, Comparator, Outcome, Time, Setting) framework [20] and quality assessment information. The data extraction was conducted by trained research assistants with oversight from three senior research team members (K.D, S.L., V.T.).

## 3. Results

**Figure 1:**
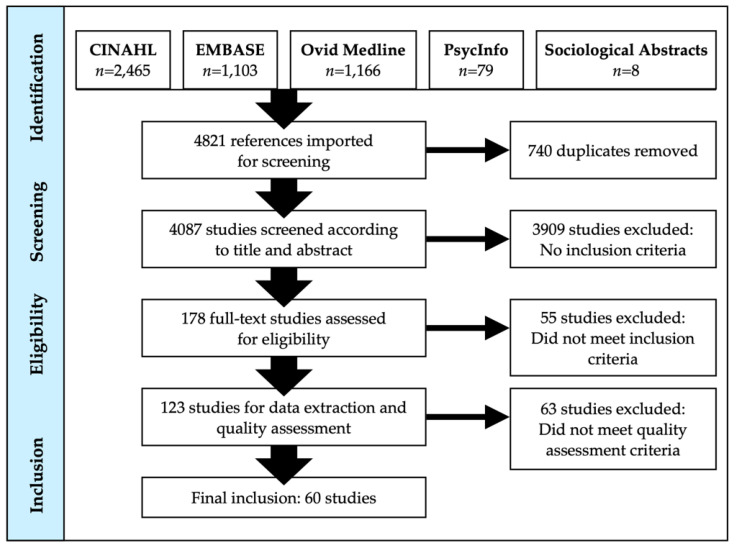
PRISMA diagram of English literature search results.

**Figure 2:**
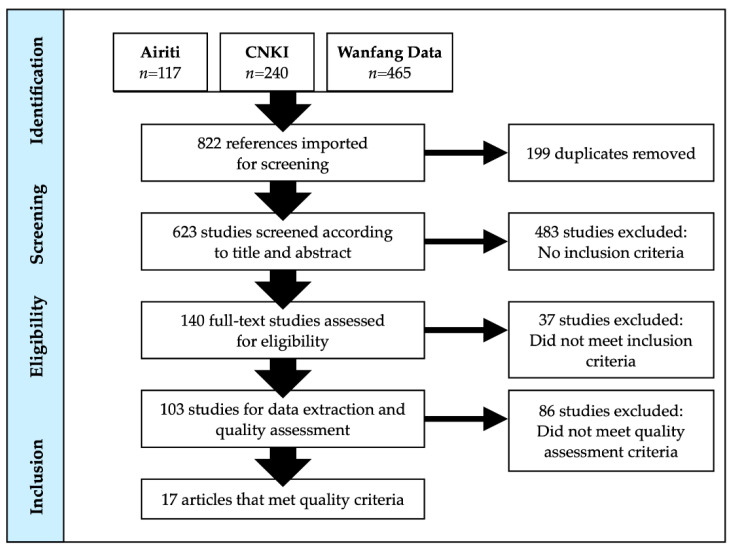
PRISMA diagram of Chinese-language literature search results.

The results of the literature searches are outlined in Figure 1 (English) and Figure 2 (Chinese). Of the 4821 English-language references imported to Covidence for screening, 740 duplicates were removed. After abstract and title screening, 178 studies were subsequently selected to undergo full-text screening. About one-third (*n* = 55) of the articles did not meet the inclusion criteria. The number of studies that underwent quality assessment was 123 and slightly more than 50% were excluded as they provided too limited information pertaining to the research questions. A total of 60 English-language articles underwent extraction.

For the Chinese-language literature search, a total of 822 titles and abstracts (Airiti Library *n* = 117; CNKI *n* = 240; Wanfang Data = 465) were located. Of these, 199 were duplicates. The 623 unique titles and abstracts were independently screened by two reviewers. When there was disagreement regarding inclusion/exclusion between two reviewers, a senior researcher (K.D., S.L.) reviewed the title and abstract and made the final decision. 140 full texts warranted full-text review, and 17 studies met the inclusion criteria and were included in the study.

The studies originated from 17 countries. Most studies were from the United States (*n* = 18; 28%), followed by China (*n* = 8; 12%), Italy (*n* = 7; 11%), Spain (*n* = 5; 8%), Canada (*n* = 4, 6%), and India (*n* = 3, 5%). Other countries included Germany, Iran, Poland, Egypt, Ukraine, France, Australia, Brazil, Ireland, and Greece. The data extraction tables summarizing the information derived from the 77 articles are located in Supplementary Tables S2 and S3.

### 3.1. Results Relevant to Scoping Review Question 1

Question: What mental health conditions and substance use risk factors are related to the COVID-19 pandemic among populations with chronic physical health conditions who are at risk of contracting COVID-19 and having severe symptoms?

In the review literature, increased levels of substance use were associated with unexpected unemployment, social distancing, and quarantine during COVID-19 [21]. Anxiety and depression were the most commonly reported mental health conditions for individuals with chronic health conditions during COVID-19 [22,23,24,25,26,27]. The following sections highlight literature findings of the most common mental health conditions (e.g., anxiety, depression) and substance use risk factors associated with specific physical comorbidities. As demonstrated in the following section, increased levels of adverse mental health conditions were primarily associated with access to needed resources (e.g., health care team support); control or lack of control (e.g., ability to adhere to treatment regimen); or concern with contracting COVID-19.

#### 3.1.1. Mental Health Conditions

##### Individuals with Cancer

In China, Zhao et al. [28] conducted a study centered on the psychological impact of the pandemic for individuals with a cancer diagnosis. The report indicated that 70% of the participants diagnosed with any type of cancer had some level of anxiety [28]. The key contributing factors to anxiety were female gender, being single, receiving in-hospital care, and lack of knowledge about infection prevention measures (*p* < 0.05) [28]. In another Chinese study by Chen et al. [29], researchers sampled individuals with cancerous tumors from five hospitals in Guangdong Province. Patients’ mental health was assessed using the Kessler Psychological Distress Scale (K-10). Of the 189 participants, 51% exhibited signs of psychological distress [29]. The researchers found a significant positive association between COVID-19 disruption of patients’ surgery or treatment schedules and their distress scores. Almost one-quarter (23%) of participants reported that they could not see a doctor as frequently as usual [29].

Xu et al.’s [30] cross-sectional survey of 368 individuals with lung cancer from 25 provinces in China found similar results. Between 40 and 75% of survey respondents reported nervousness, anxiety, and/or quality of sleep disruption [30]. Interestingly, reports of anxiety among respondents were not related to fear of dying from COVID-19 [30]. The mental health measures that were used in this study were not clearly indicated.

In an Italian retrospective cohort study that focused on anxiety among women accepting treatment for breast cancer in the context of the COVID-19 pandemic [31], there were significantly higher rates of refusal for procedures (*p* = 0.028) and surgeries (*p* = 0.0065) in the COVID-19 period when compared to pre-COVID-19. The researchers hypothesized that these refusals were due to women’s concerns of contracting COVID-19 [31]. In this study, there was no specified measure used to assess anxiety.

##### Individuals with Diabetes

In India, Nachimuthu et al. [24] conducted an online pilot survey to study how people with type 1 and type 2 diabetes were coping with their health conditions during the COVID-19 lockdown. Among 100 participants, 92% had type 2 diabetes, over 50% were males and over 65 years of age, and 8% had other physical complications, such as cardiac or kidney disease. Approximately two-thirds (65%) of the participants who were taking oral medications and insulin were not testing their blood glucose levels regularly. However, 80% of the sample reported that they were exercising regularly and following dietary recommendations. Almost half of the respondents (40%) were anxious about the COVID-19 situation, and, at the time of this study, 73% believed their current situation would improve in the near future. Furthermore, about 8% of the study participants had cardiac and kidney complications; details about their mental health history were not reported.

A study conducted in China [32] examined barriers during the pandemic for maintaining a healthy mental state among 75 individuals with diabetes. The questionnaire the participants responded to included Zung’s Self-Rating Anxiety Scale and Self-Rating Depression Scale. Based on regression analysis, factors which contributed to anxiety and depression included area of residence, presence of diagnosed cases of COVID-19 of people near the participant, whether there is sufficient mask supply, and availability of diabetes medication. An individuals’ ability to follow their medical doctor’s advice also impacted their mental state. When those who did not appear to follow their treatment plan were compared to those who did, depressive symptomatology was reported to be significantly higher (*p* < 0.05) [32].

##### Individuals with Epilepsy

In a Chinese case-control study by Hao et al. [33], predictors of psychological distress, as measured by the 6-item Kessler Psychological Distress Scale, were compared for individuals with epilepsy (*n* = 252) and controls without epilepsy (*n* = 252). Individuals with epilepsy had significantly higher distress scores than controls and spent significantly more time following the news about COVID-19 (*p*’s < 0.001). Results of multivariable logistic regression analysis identified two independent predictors of psychological distress: time spent paying attention to media reports related to COVID-19 (odds ratio (OR) = 1.17, 95% CI 1.07–1.28) and diagnosis of drug-resistant epilepsy (OR = 0.28, 95% CI 0.13–0.62).

##### Individuals with Obesity

In Poland, an online survey was conducted with patients who were pre- (*n* = 258) and post-operative (*n* = 548) for bariatric surgery [34]. All respondents had physical health conditions, such as insulin resistance (*n* = 224), type 2 diabetes (*n* = 93), obstructive sleep apnea (*n* = 63), arterial hypertension (*n* = 265), dyslipidemia (*n* = 68), or arthritis/joint pain (*n* = 272). Almost three-quarters of respondents also reported high levels of anxiety (74.5%). The majority of respondents (72.3%) were aware that obesity was an important risk factor for heightened severity from COVID-19 infection. Despite this knowledge, about one-third (29.5%) of respondents had experienced weight gain with higher proportions among those who were pre-operative versus post-operative (43.8% vs. 22.7%; *p* < 0.001). Only 20.9% of all respondents had ongoing access to direct care from the bariatric team. Although remote access was reportedly available to 67% of the respondents, the researchers surmised that access challenges adversely influenced eating habits, levels of physical exercise, and psychological distress, leading to deterioration of sustained weight loss for both populations [34]. Similarly, a review by Sockalingam et al. [35] reported that individuals undergoing bariatric surgery experienced increased levels of emotional distress. In this population, increased stress can exacerbate eating psychopathology, adversely impact treatment adherence, and contribute to poorer long-term health outcomes [35].

##### Individuals with Parkinson’s Disease

A telephone survey conducted in Germany reported higher levels of anxiety (25.5%) in 99 individuals with Parkinson’s disease (PD) compared to 21 controls matched by age and gender (4.8%) [36]. The researchers found a significant positive correlation between severity of anxiety and fear of COVID-19 diagnosis among those with PD. Higher anxiety levels were also reported among individuals with PD who were concerned about drug availability during the lockdown as well as those with other chronic medical conditions. Among respondents with PD, stress-related psychiatric symptoms, including anxiety, were 30–40% more common than in the general population which was thought to be due to motor and cognitive inflexibility as well as reduced capacity to readily adapt to additional stressors from COVID-19 [36].

##### Individuals with Respiratory Conditions

A small study conducted in China [37] studied a small number of individuals (*n* = 8) with a history of respiratory problems. The whole sample showed some evidence of post-traumatic stress disorder (PTSD) related to fear of contracting COVID-19. The sleep quality of all these individuals was adversely affected, manifesting as night-time sleep difficulties and early awakening. Disrupted sleep cycles are associated with symptoms of anxiety, PTSD, and depression, with severe cases leading to suicidal thoughts [37].

#### 3.1.2. Substance Use

##### Individuals with Risk Factors for Substance Use

Only two studies were located that discussed substance use risk factors. A phone survey in Ukraine [38] examined the impact of social supports among older adults with HIV and substance use disorder (*n* = 123) during the COVID-19 lockdown. The 123 respondents maintained their treatments throughout the COVID-19 lockdown; however, they still had anxiety about the availability of treatment services. Almost two-thirds (61%) of respondents reported they had no one available as a treatment supporter. The authors noted that social support was critical for avoiding treatment interruptions [38].

In a cross-sectional study conducted in Poland [39], it was reported that individuals with substance use disorder and higher BMIs were adversely affected by quarantine during COVID-19. Approximately 43% of individuals with higher BMIs increased their food intake; 51.8% did more snacking between meals; 14.6% consumed more alcohol; 45.2% reported increased smoking [39]. Among the 14.6% who reported increased alcohol use, the proportion was higher among those who reported they had an alcohol addiction. The authors noted that future research should try to determine whether the COVID-19-related lockdown has resulted in long-term reinforcement of adverse dietary habits and associated health issues, such as substance use [39].

### 3.2. Results Relevant to Scoping Review Question 2

Question: What are effective health promotion, primary prevention, screening, and treatment interventions to enhance mental health outcomes and to reduce risk of substance use for populations with chronic physical health conditions who are at risk of contracting COVID-19 and having severe symptoms?

#### 3.2.1. Individuals with Various Physical Health Conditions

In a study that examined adults (*n* = 269) who had a disability or chronic condition [40], it was reported that perceived stress related to COVID-19 was positively correlated with self-distraction, denial, substance use, behavioral disengagement, venting, planning, religion, and self-blame. Results from the hierarchical regression analysis indicated that active coping, denial, use of emotional support, humor, religion, and self-blame were associated with well-being after controlling for demographic and psychological variables. Results of this study suggest that focusing efforts on positive coping may help mitigate stress associated with the COVID-19 pandemic among those with chronic conditions and disabilities [40].

In another study by Germani et al. [41], 1101 emerging adults (18–29 years old) were surveyed about their mental health responses to COVID-19. The survey included measures of the cultural dimensions on psychological maladjustment. Of the total sample, at least 31% had pre-existing physical or mental health conditions. The results indicated that perceptions of the self as part of a larger collective where all members of the collective are the same and equal [42] were associated with reduced emotional and behavioral difficulties, anxiety, and stress [41].

#### 3.2.2. Health Promotion and Primary to Tertiary Prevention

Within the extracted literature, many studies discussed the use of telehealth strategies and online platforms to maintain access and communications with individuals. Mental health promotion strategies focused on decreasing anxiety and depression by ensuring access to disease-specific care and to psychological supports. In some instances, the importance of the family was highlighted. Some Chinese studies emphasized overarching prevention programs, facilitated by experts (e.g., psychologists, psychiatrists), that include content and resources on mental health, life guidance, and personality development. Other Chinese papers and studies from other countries focused on specific interventions, such as cognitive behavioral therapy. Equity-seeking populations (e.g., racialized communities) have inequitable access to the social determinants of health, predisposing them to physical and mental health conditions—and to COVID-19. Community-based integrated physical and social care services are particularly important sources of health care support for vulnerable individuals. Examples follow of mental health promotion and prevention strategies for specific populations.

##### Individuals Living with Cancer

An article by the International Geriatric Radiotherapy Group [43] discussed how to prevent depression and anxiety among older adults with cancer. Recommendations included providing personalized care that is based on assessment of physical health and socio-economic status, social services such as patient navigators, close monitoring via phone calls and telecommunications, and helping families provide psychological support [43].

A survey of people diagnosed with genitourinary cancers in Germany aimed to examine their perspectives on telehealth use during and after the pandemic [44]. Of the 101 sampled, 92 responded to the questionnaire. Among the sample were individuals who also had underlying conditions such as cardiac disease, diabetes, renal disease, obesity, pulmonary disease, or a compromised immune system. For most participants, their anxiety about cancer superseded that of contracting COVID-19 infection (*p* < 0.001). Most opposed interruptions to their treatment and highly rated the use of telehealth during the crisis, but they preferred it not continue after the pandemic. Most did not believe they were more susceptible to COVID-19 compared to the general population. This study suggests that telehealth interventions are valuable in helping provide mental health services in a pandemic; however, there use otherwise needs to be further evaluated [44].

In a paper from China studying individuals with cancerous tumors, a 7-step prevention strategy to protect people from psychological stress was used [45]. The strategy is based on three-over-arching components: mental health education, life guidance, and personality development. The steps are: [45]:

i. accept that distress is a normal response to cancer and the COVID-19 epidemic;
ii. trust the doctor and build a harmonious doctor-patient relationship;
iii. maintain a stable life routine; be positive and optimistic;
iv. communicate with relatives and friends; do not conceal your health status;
v. arrange appropriate recreational activities every day and perform aerobic exercises when your physical condition allows. Recommended activities include yoga and traditional Chinese-based aerobic activities such as Tai Chi and Baduan Jin to balance qi (circulating life force);
vi. establish a healthy sleep cycle and avoid using a mobile phone in bed;
vii. when self-regulation cannot relieve anxiety, depression, and other emotions, seek professional help from a psychiatrist or psychologist.

Those who proposed the 7-step strategy [45], were not optimistic about the prevention and treatment of mental health symptoms in some types of cancers, such as hematological cancer.

A Chinese paper [46] reported the use of an online hospital platform for providing mental health education led by psychology experts. Oncology and infection specialists were also available during online counseling sessions to answer questions about physical health and to provide the latest, accurate updates on COVID-19 [46]. No specific outcomes were reported about their interventions.

Another Chinese study reviewed the merits of managing cancer patients’ psychological distress during COVID-19 using the following interventions: cognitive behavioral therapy (CBT), mindfulness-based stress reduction (MBSR), and narrative therapy [47]. The authors reported that CBT intervention during the denial stage after cancer tumor diagnosis helped prevent maladaptive emotional, behavioral, and physical responses related to distorted beliefs [47]. Cognitive behavioral therapy, which challenges distorted beliefs, is associated with reductions in depression and stress responses and with increased positive coping styles [47]. Mindfulness-based stress reduction directs the person’s attention to the present situation and development of an accepting attitude. This approach decreases anxiety and depression symptomatology for patients with malignant tumors. Lastly, the authors described narrative therapy as a type of psychotherapy that helps foster a sense of security and inclusion. Overall, the authors supported the use of these therapies through hotline assistance and individual and group counseling [47].

In Italy, Vanni et al. [31] conducted a retrospective study to observe the refusal rate of breast cancer treatment in female breast cancer patients aged 45–80 before and after the COVID-19 pandemic. At the start of the pandemic, the refusal rate increased due to fear of COVID-19. The authors recommended psychological support to address COVID-19-related anxiety and consultations with surgical oncologists to discuss the risks associated with refusing treatment (such as that for advanced breast cancer) [31].

##### Individuals Living with Other Chronic Health Conditions

In order to effectively reduce psychological pressures in individuals with chronic kidney disease who need dialysis, a Wuhan hospital established a “five in one” mental health framework [48] that included the following components:

i. strengthen health education related to COVID-19 through the WeChat online platform;
ii. increase information transparency to reduce anxiety, especially to correct misunderstandings about the pandemic from unreliable sources;
iii. strengthen the support offered by the doctor and family members. By receiving external support, patients may be better able to face the challenges of the pandemic;
iv. provide nutrition guidance. Proteins are especially crucial, as the process of dialysis leads to a loss of proteins;
v. guide individuals to use the professional mental health channel, as established by the hospital through the WeChat online platform.

In a descriptive study by Zhang et al. [32], the mental health of people with diabetes in China focused on the importance of accurate, timely COVID-19 information tailored to those living at home. The researchers made reference to an online portal for mental health counseling. In addition to counseling, this online service provided timely, accurate information about COVID-19, to alleviate distress for those with diabetes [32].

During the pandemic, older adults with chronic physical health conditions (e.g., diabetes mellitus, cardiovascular disease, immunocompromised, chronic lung conditions, renal disease) had higher morbidity and mortality rates and significant adverse psychosocial effects due to isolation and quarantine [49]. Common recommendations to address isolation for this population were telehealth technologies to conduct virtual clinician assessment of physical and mental health risk factors and to provide prevention/promotion education (e.g., healthy dietary habits, stress reduction) and psychosocial supports [50]. In the US [51], social workers virtually collaborated with clients to address loneliness and chronic stress and to develop chronic disease self-management skills. In China, available family members were the primary care providers for older adults with physical health conditions at home [52]. Chinese nurses performed ongoing virtual/in-person assessments of older adults’ physical care needs and family members’ capacity to safely provide basic care, while both social workers [53] and nurses [52] conducted periodic virtual and/or in-person mental health check-ins with at-home older adults, particularly solitary adults. During the pandemic, an overarching goal was to keep this population of seniors safe in their homes [52].

##### Individuals Living with Complications from COVID-19

Complications due to COVID-19 are beginning to garner more attention. Ceravolo et al. [54] conducted a systematic rapid “living” review to determine the rehabilitation needs of individuals living with complications due to COVID-19 infection. Restricted mobility due to COVID-19 infection or lockdown warrant early rehabilitation (e.g., physiotherapy, exercise programs) and access to telehealth and telerehabilitation programs for individuals with complications. Of note, telehealth programs should address physical and “cognitive” rehabilitation options based on needs assessments of individuals living with cardiac complications and disabilities resulting from COVID-19 [54].

In a consensus statement on rehabilitation needs of long-COVID survivors, the most evidence-based recommendations (1a on the Oxford Levels of Evidence scale) included referrals to psychological services, trauma-focused cognitive behavioral therapy, and active monitoring of those with subclinical psychological symptoms [55]. Lower quality recommendations (level 5 on the Oxford Levels of Evidence scale) included providing effective patient communication and remote social contact and reviewing mood and well-being of patients without psychological symptoms. A similar published clinical practice guideline [55] similarly recommended that primary care physicians should conduct social prescribing for individuals experiencing adverse psychological events during COVID-19. The evidence for social prescribing was not reported, but social prescribing enables physicians to connect individuals to community supports and activities geared towards social connectedness and mental well-being (e.g., yoga, art classes, virtual choir, online drawing classes).

A randomized, controlled study that examined depression and anxiety in 26 Chinese individuals with COVID-19 (18–65 years old) was conducted [56]. The participants who were in an isolation ward were initially screened for psychological distress and followed for 2 weeks in an isolation ward. These patients all had pre-existing physical health conditions (four had hypertension, two had liver disease, and one each had gastric ulcer, coronary heart disease, or AIDS) [56]. The intervention group (*n* = 13) received a self-help internet-based intervention of breath relaxation training, mindfulness (body scan), “refuge” skills, and the butterfly hug method. Intervention subjects listened to 50-min audio recordings via their mobile phones and followed intervention training instructions at a fixed time every day for two weeks. Those in the control received physical supportive care (*n* = 13). At baseline, there were no significant differences between the two groups for age, gender, severity of illness, and anxiety and depression scores. At the conclusion of the study, there were no main or interactive effects for age, gender, or severity of illness. At the end of the first week and the second week, the intervention group had significantly lower depression and anxiety scores compared to the control group [56].

##### Individuals Living with Dementia

In China, practitioners were advised to create detailed contingency plans for virtual care and support of individuals with dementia and their families [57]. Virtual dementia care education for practitioners was also available through an online platform.

For nursing home residents with Alzheimer’s, US researchers investigated the use of FaceTime interactions with family members to reduce behavioral problems [58]. Residents enjoyed FaceTime sessions, and they had improved appetites and decreased levels of anxiety and agitation. Families reported that FaceTime sessions created a sense of connectedness for them. Spanish researchers [59] trialed a television-based assistive integrated service, TV-AssistDem, to support community-dwelling older adults living with mild dementia, mild cognitive impairment, and/or anxiety. TV-AssistDem includes video interactions with health care providers and can provide services such as reminders, cognitive stimulation, and health monitoring/data transmission. No evaluative data were available in this descriptive paper [59].

Brown et al. [60] recommended virtual care telehealth programs for older adults at home with Alzheimer’s disease and related dementias. They acknowledged, however, that virtual diagnosis and care via telephone or videoconferencing may not be sufficient to properly complete comprehensive physical and cognitive examinations for diagnostic purposes.

##### Equity-Seeking Individuals with Pre-Existing Physical Health Conditions

Systemic racial discrimination and stigma, particularly within communities of color (e.g., African American and Hispanic communities), increase vulnerability to the negative effects of COVID-19 [54,61]. Fortuna et al. [62] reported data from the Centers for Disease Control and Prevention demonstrating that Black Americans accounted for 34% of confirmed COVID-19 cases, despite only comprising 13% of the total US population. African Americans were disproportionally at higher risk of COVID-19 infection due to pre-existing vascular and respiratory-related diseases and they encountered medical biases with respect to testing and treatment for COVID-19 [61]. For populations with physical and social care needs, integrated services in local communities are necessary to ensure equitable access [62]. Features of effective integrated care include collaborative leadership and governance between services and community members—to effectively empower and engage end users [62].

Access to HIV testing and treatment were adversely affected during the pandemic, particularly for equity-seeking populations [63,64,65]. Telemedicine initiatives for mental health assessments and HIV-related care were believed to be beneficial services for individuals with high-speed internet. Individuals with access issues to the social determinants of health, however, often had income barriers that challenged their capacity to engage in telehealth services—adversely affecting treatment adherence [63,64,65]. Rogers et al. [64] highlighted COVID-19 service adaptations for two US sexually transmitted infection (STI) clinics that provided evidence-based psychotherapy, HIV testing, substance use treatment, and other services geared towards sexual minority individuals. Virtual service transition failed for individuals who lacked stable housing and technology. To improve service access, the STI clinics eventually employed a more effective peer-based recovery model with peer coaches who provided flexible meeting times in convenient locations [64].

In a US survey study by Sanchez et al. [66], researchers assessed the COVID-19 impacts on the sexual health of men who have sex with men (MSM) who were diagnosed with substance use disorder and HIV. Most survey respondents reported increased anxiety (73.4%). Several participants identified problems with basic resource needs (e.g., difficulty buying food, paying rent). These problems were more likely in younger participants (ages 15–24) who also reported increased recreational drug use and alcohol consumption compared to MSM respondents 25 years and older. Younger participants also reported more problems with access to testing and treatment related to HIV and sexually transmitted infections. The authors’ primary recommendation was better telehealth services for physical and mental health needs [66].

##### Individuals Living with Substance Use

Ahmed et al. [67] conducted a survey of individuals (14–68 years old) in Hubei, China with reported mental health concerns (depression, anxiety) and excess alcohol consumption. In this cross-sectional study conducted during the pandemic, levels of anxiety and depression increased, mental well-being decreased, and alcohol consumption stayed relatively the same. The authors provided several recommendations, such as restricted media exposure about COVID-19, access to online counseling for screening and treatment, proactive health care provider psychological training for vulnerable populations, and strategic planning for virtual rehabilitation services [67].

In the US, Da et al. [68] examined interventions during the COVID-19 pandemic for addressing the physical and mental health needs of individuals with alcohol use disorder (AUD) and alcohol-associated liver disease (ALD). Recommended interventions were use of telehealth and secure messaging services for 24/7 care related to alcohol counseling, screening, and surveillance for those at risk of relapse and addiction treatment. Mobile applications, such as EncephalApp, were instituted to assess cognitive function related to hepatic encephalopathy and to augment telehealth visits [69]. A notable recommendation was proactive planning for specialized, multidisciplinary integrated treatment centers to prepare for the expected increase in AUD, ALD, and the adverse physical and mental health effects associated with them [69].

## 4. Discussion

The main objective of the scoping review was to capture evidence about what mental health promotion, prevention, and intervention strategies may reduce adverse mental health conditions and substance use risk factors related to the COVID-19 pandemic among populations with chronic physical health conditions who are at risk of contracting COVID-19 and having severe symptoms.

Much of the published literature highlighted mental health implications, including anxiety, depression, distress, disordered eating, and substance use, which occurred in individuals with chronic conditions such as cancer, cardiovascular disease, dementia, diabetes, HIV, and Parkinson’s disease. The fear of contracting COVID-19 among those who knew they were more susceptible to infection and the associated mental health impacts interfered with adherence to treatment plans. Several promising practices were described but not formally evaluated. The Chinese-language literature tended to discuss more self-help and alternative measures, such as the butterfly hug technique, to manage mental health. The following accumulated evidence from the scoping review focuses on mental health promotion across the health care continuum, future research, and means to organize health and social services that are responsive to public health crises.

### 4.1. Factors Contributing to Mental Health and Well-Being

**Figure 3:**
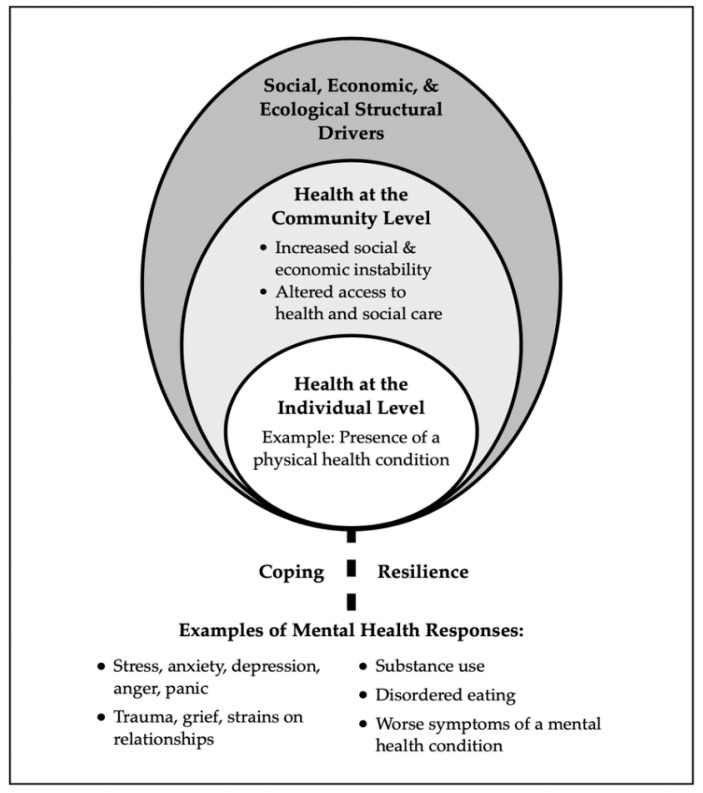
Factors impacting mental health for those with physical health conditions that present risk of severe COVID-19 infection.

In the surveys about mental health responses to COVID-19 which compared those with and without chronic conditions, higher levels of distress tended to be reported among those with a chronic physical health condition [56,70]. There are multiple intersecting individual, community, and societal level factors that may contribute to mental health and well-being among those who have a chronic physical health condition that places them at risk of severe COVID-19 infection (Figure 3). First, as shown in Figure 3, there are structural level drivers that include ecological (e.g., food supply and availability), economic (e.g., income supports), and social factors (e.g., mental health stigma) that affect community and individual level health. At the community level, the pandemic’s effects include outcomes such as increasing economic instability and social isolation as well as decreasing access to health and social care services. It is well established that chronic physical health conditions such as obesity, diabetes, cancer, cardiovascular disease, chronic respiratory diseases, chronic kidney and liver diseases, autoimmune conditions, HIV, hepatitis, and frailty present risk for severe COVID-19 infection [4]. Furthermore, mental health problems and conditions such as major depression, schizophrenia spectrum conditions, psychosis, anxiety disorders, and bipolar affective disorders are commonly comorbid among various chronic physical health conditions, including diabetes, cancer, cardiovascular disease, chronic respiratory diseases, arthritis, and inflammatory bowel disorders [71,72,73,74,75,76,77]. The co-occurrence of physical and mental health issues results from an interplay of biological factors (e.g., increased inflammatory response), genetic predisposition, behavioral factors such as poor diet, physical inactivity, or substance use, and psychosocial factors such as illness experience [78]. These factors impact an individual’s coping and resilience [79] and increase the likelihood that the person living with a physical health condition will have mental health responses such as anxiety, depression, or substance use. Alternatively, if they have a co-existing mental health condition, their symptoms may worsen [5,6].

### 4.2. Mental Health Promotion and Policy

Of the extracted studies, there did not appear to be explicit discussion about health and social policies aimed at modifiable targets, such as income, to help prevent or manage mental health concerns during the pandemic. As Horton suggests [80], SARS-CoV-2 interacts with an array of non-communicable diseases and these conditions cluster within subpopulations according to patterns of deeply embedded inequality. Furthermore, the nature of the COVID-19 threat, which includes interactions of biological, psychological, and social factors that impact health, points to the need for proactive and nuanced approaches to protect the health of communities. Certain health and social policies may help to create supportive environments for mental health promotion across different populations. Some examples of policy areas that elevate population mental health include healthy child development, mental health screening, the social determinants of health (e.g., income, education, literacy) and socio-economic inequities [81,82,83]. In addition, directives towards ensuring consistent and appropriate access to mental health services, including specialist services, can help maintain mental health and well-being, especially during public health crises.

Some literature has highlighted policies aimed at substance use during the pandemic [84]. For example, psychosocial crises may trigger alcohol abuse that in turn may contribute to impulsivity, aggressiveness, loneliness, and hopelessness [85]. Strategies to monitor alcohol consumption during pandemics are recommended with restriction of access as needed to reduce risk of abuse. Furthermore, policy targets aimed at reducing risk factors for substance use, such as unemployment [86,87], may help to prevent substance abuse during public health crises.

### 4.3. Health Education and Literacy

One of the main consequences of the COVID-19 pandemic has been the dissemination of rumors and health misinformation through media and social networks [88] which has the potential to lead to reactions that can worsen mental health [89,90]. As identified by researchers and decision makers, parallel to the current pandemic has been a massive “infodemic” of rapidly spreading misinformation through social media platforms and other outlets [89]; this needs to be addressed by the public health community who can educate and support social and conventional media to positively deliver information from health, medical, and scientific communities [90].

Individual health literacy is defined as the extent to which people have the capacity to obtain, process, and understand basic health information and services needed to make appropriate health decisions [91]. As suggested by different investigators [36,39], low health literacy may have contributed to adverse mental health responses during the COVID-19 pandemic. Lower levels of health literacy are associated with both poorer physical and mental health [91,92]. Health literacy policies and programs, such as aids to improve self-management of chronic diseases, should target vulnerable groups, such as those with lower levels of education, lower incomes, and low language proficiency to ensure equitable access to vital information [92].

A barrier to mental health that was not identified in the studies, but which has been reported elsewhere [93], are the stigmas that can occur with both physical and mental health conditions. Physical health conditions such as obesity, lung cancer, and arthritis may be prone to associated stigmas [94], with reported negative effects including anxiety [95], stress [96], depression [97], and reduced self-esteem/self-efficacy [98]. Studies also suggest that, as physical health worsens, the risk of experiencing discrimination increases [99]. Some investigators have also pointed to the ageist rhetoric which has been a dominant theme during the COVID-19 pandemic [99]. Public health education campaigns aimed at reducing stigmas can help shape more positive attitudes about illness and aging and increase the quality of life of those targeted by stigma [99,100].

### 4.4. Health and Social Care Delivery

Prior to the pandemic, it was identified that there was a need for more integrated physical, mental, and social health care services. Integrated services are associated with reduced morbidity, premature death prevention, improved service access and service transitions across health care, enhanced service sustainability and stability, and improved community health [101]. In many countries, the collaborative care model is considered best practice for optimal physical and mental health integration. Collaborative care models have supported important innovations associated with mental health promotion such as crisis helplines to help identify and intervene in emerging psychosocial crises [102], expanded social worker roles and the inclusion of case managers to help monitor individuals at risk for poor mental health [102], and social prescribing practices that connect individuals to community-based services and activities, including meditation and yoga classes [103].

Collaborative practice is broadly defined as a multi-professional approach of shared communications and decision-making with patients at the center of care. Collaborative models of care are typically community-based primary points of service contact for end users [104,105]. This evidence-based model of care has faced implementation and sustainability challenges due to lack of adequate resources. Lack of resources to support collaborative care became apparent during the pandemic, when many community-based services closed down due to fear of COVID-19 outbreaks [106]. Achieving capacity for more resilient and flexible collaborative care will require increases in human resources and enhancements in technology that facilitate sharing of information [102].

Strategies that facilitate collaborative care and integrated health and social care include [107,108,109]:

- registries of people with complex needs to track preventative care, disease/illness management, and referrals to secondary and tertiary care services;
- health care system navigation supports and shared decision-making approaches;
- competencies among practitioners to deliver high-quality health care to people with mental health problems;
- training practitioners in the recovery model, including stigma, discrimination, and trauma-informed care;
- evidence-based mental health screening guidelines with accompanying management pathways.

Another alternative model of care is group medical visits for individuals who share the same chronic condition. Usually after an initial screening visit with a practitioner, individuals are invited to attend group sessions to receive care, education, and advice within a supportive group environment [110]. These visits may also include other practitioners such as nurses, dieticians, and social workers. Group medical visits are cost-effective alternatives to individual psychiatric outpatient care or mental health care for individuals with moderate or severe mental illness [111]. This model of care has been delivered through secured videoconferencing, however, to the best of our knowledge, the effectiveness of a virtual approach to group medical visits has not been evaluated.

Patient navigator systems that guide individuals through the health system, according to their treatment needs [112], may be effective mechanisms to support mental health. Patient navigators may be nurses, social workers, lay health workers, or peers. Specific tasks of the navigators may include providing disease and health system education, addressing financial barriers, conducting care coordination, and providing emotional support [113]. These programs have been used for people with HIV/AIDS, cardiovascular disease, chronic kidney disease, dementia, and individuals with comorbidities [113]. One 9-month RCT of 432 women with a chronic health condition assigned participants to either a public health nurse case manager or a wait-list control group. From study onset to completion, the intervention group reported significant decreases in depression scores and improvements in functional status compared to the control group (*p* = 0.016) [113]. This service delivery model may prove useful during times of disease outbreaks where navigators can assist people through the health system, potentially through the use of digital applications.

The use of different wireless technologies to support the achievement of health objectives [114], known as m-health, has been discussed in some of the papers of this scoping review [44,58,62,67]. Most discussions focused on resources and services provided via the internet, videoconferencing, or mobile apps that monitor both physical and mental health symptoms. Enhancing delivery of mental health care using m-health applications can also increase the effectiveness of paraprofessionals, peer helpers, and mutual aid organizations [115] and augment telehealth visits [116].

One of the most widely investigated m-health applications used in the context of crises such as wars and natural disasters has been PTSD Coach, which may have applications in infectious disease pandemics [116,117,118,119,120,121]. An RCT of PTSD Coach was conducted prior to the COVID-19 pandemic with 120 community trauma survivors [121] who reported significant reductions in PTSD and depression severity and improved psychosocial functioning relative to wait-list controls. These study results occurred after three months of PTSD Coach app use by the intervention group [121]. A variety of web-based interventions for combat veterans experiencing post-traumatic stress difficulties have successfully reduced PTSD symptoms, depression, and/or alcohol use [119,120,121]. Ideally, such m-applications should include features such as monitoring of wellness, cognitions, and emotions [121]. Digital reality applications, such as virtual reality (VR), are being explored to help improve mental health. For example, at Cedars-Sinai Medical Center in Los Angeles, VR is being applied during the COVID-19 pandemic to deliver mind-body treatments in three-dimensional worlds for people in their homes [122].

Although m-health applications are recognized as viable alternatives to health care delivery, particularly during the pandemic, equitable access remains an ongoing concern. Certain populations, such as those from racial and ethnic minority groups, have been disproportionately burdened by disparities in digital access during the pandemic, and these may intersect with other forms of structural disadvantage and exacerbate the consequences of COVID-19 [123]. Future policy considerations must aim to eliminate digital health inequities by increasing access to broadband internet and improving digital literacy [123].

### 4.5. Mental Health Screening and Assessment

Mental health screening among those with physical health conditions is a recommended standard of practice [124], particularly in the context of the COVID-19 pandemic. There is considerable heterogeneity among the measures being employed, some of which have also not been validated for use in people with different health conditions. Tools to assess mental health for those with chronic physical health conditions and who are at risk of communicable disease should account for symptoms (e.g., fatigue), stress, quality of life, risk factors, and protective factors. Brief screening tools, such as the two-item Patient Health Questionnaire (PHQ-2), may be feasible for use during virtual visits or as part of testing for COVID-19. If more comprehensive assessment is needed, people can be scheduled for an assessment with standardized instruments validated for their particular circumstances (e.g., health condition) [124].

Gatekeeper training involves training key people, such as first responders, to identify individuals experiencing mental health issues [125]. In the context of the pandemic, it may be helpful to train gatekeepers in the general population (e.g., teachers). Programs such as Mental Health First Aid—Australia incorporate gatekeeper training that has been effective for improving knowledge and attitudes and promoting helping behaviors towards adults with mental health problems [125].

### 4.6. Psychological, Lifestyle, and Peer Approaches

Scoping review recommendations to promote mental health during the pandemic included self-care and some standard mental health interventions, such as cognitive behavioral therapy (CBT). Brief CBT interventions focused on the identification of warning signs, coping skills, social support, professional help, and crisis planning have been effective in preventing suicidal thoughts and behavior [126]. While CBT is considered a cornerstone of mental health care, the literature does not demonstrate its efficacy for individuals with different chronic physical health conditions.

Similar to the psychosocial intervention literature, there was limited discussion in review papers about lifestyle and peer-based interventions to improve mental health outcomes. Lifestyle interventions such as physical activity, diet, and mindfulness-based meditation techniques may promote mental health when used as adjuncts to evidence-based physical care regimens for individuals with chronic physical health conditions [127]. Peer support approaches is another recommended model for mental health care. Evidence has shown it promotes early help-seeking, prevents mental health-related hospitalizations, reduces extended hospital stays, and eases demands for more specialized mental health professionals [128].

### 4.7. Strengths and Limitations of the Scoping Review

This review contributes to an understanding of mental health responses among people with physical health conditions that present risk of having severe infection from COVID-19. It also highlighted interventions that may help prevent and manage mental health responses in these populations. The use of a scoping review provided broad coverage of the literature highlighting the main issues that can help provide the information needed for health policy, clinical practice, and future research. Although many literature sources were searched, due to the rapid timeline for the review, we were unable to continuously search for and integrate new peer-reviewed and gray literature sources in English or Chinese.

## 5. Conclusions

There are various interventions that may foster mental health for those with chronic physical health conditions in the context of a pandemic; however, an evidence base outlining their effectiveness is lacking. A better understanding of the shared etiology of mental and physical health will lead to more targeted and personalized approaches to care. In addition, more knowledge about integrated health and social care implementation and outcomes are needed. Many health-related innovations, such as telehealth technologies, were trialed during the pandemic. Further evaluation is required to determine the efficacy of these innovations, including ways to adapt and adopt them for different populations and settings, such as equity-seeking populations with barriers to the social determinants of health. This knowledge synthesis proposed many directions to take in our exploration of mental health promotion, prevention, and intervention for individuals living with chronic physical health conditions during the COVID-19 pandemic. Hopefully, this review will serve as a stepping stone for future researchers, policy-makers, practitioners, educators, and end users.

## Data Availability

Not applicable.

## Supplementary Materials

The following are available online at https://www.mdpi.com/article/10.3390/ijerph18147265/s1, Table S1: English and Chinese language data sources and search terms, Table S2: English Literature Data Extraction Tables, Table S3: Chinese Literature Data Extraction Tables.
