# Supplementary material for: Interventions to Support Mental Health among Those with Health Conditions That Present Risk for Severe Infection from Coronavirus Disease 2019 (COVID-19): A Scoping Review of English and Chinese-Language Literature
Source: Int J Environ Res Public Health. 2021 Jul 7;18(14):7265. doi: 10.3390/ijerph18147265 (PMC8303838; doi:10.3390/ijerph18147265)
Supplement: Supplementary file 1 [file ijerph-18-07265-s001.zip › ijerph-1230679-supplementary.pdf]

Supplementary Table

Table S1: English and Chinese language data sources and search terms

English Language

English Language Academic Databases	English Language Grey Literature Sources		English Language Search Terms
• MEDLINE (Ovid)<br>• Cumulative Index of Nursing and Allied Health (CINAHL)<br>• PsycInfo<br>• Sociological Abstracts<br>• EMBASE	• OCLC PapersFirst and OCLC ProceedingsFirst<br>• Action Hepatitis Canada<br>• Alzheimer’s Society of Canada<br>• Anxiety Disorders Association of Canada<br>• Asthma Canada<br>• Canadian AIDS Society<br>• Canadian Association of Psoriasis Patients<br>• Canadian Frailty Network<br>• Canadian Liver Foundation<br>• Canadian Lung Association<br>• Canadian Mental Health Association<br>• Canadian Nurses Association<br>• Canadian Psoriasis Network<br>• Canadian Psychological Association Cardiac Health Foundation of Canada<br>• Crohn’s & Colitis Canada<br>• Epilepsy Canada<br>• Equally Well<br>• Getty Publications	• Heart and Stroke Foundation of Canada<br>• International Credential Evaluation Service (ICES)<br>• Medical Psychiatry Alliance<br>• Mental Health International<br>• Mental Health Foundation<br>• MIND<br>• Mood Disorders Society of Canada<br>• Obesity Canada<br>• Obsessive and Compulsive Disorder (OCD) Canada<br>• Organization for Economic Co-operation and Development<br>• Provincial/territorial health ministries (n=13)<br>• Project Muse and Infobase<br>• Saskatchewan Health Quality Council<br>• Schizophrenia Society of Canada<br>• Te Pou Matakana Whanau<br>• University of California Press<br>• World Health Organization	Search terms related to COVID-19, mental health issues, and comorbid physical conditions (COVID-19 coronaviruses AND (Obes* or Diab* or Neoplas* or tumor or cancer or malignan* Cardiovasc* or heart* or coronary* or myocar* or stroke* or cerebrovas* or ischem* or Respir* or asthma or lung dis* Or pulmon* or chronic obstructive lung or chronic obstructive pulmonary or COPD or dyspnea or emphys* or bronchitis or Rheumatoid arthritis or systemic lupus erythematosus or multiple sclerosis or Guillain-Barre syndrome or demyelinating polyneuropathy or psoriasis or inflammatory bowel disorders or IBD or colitis or ulcerative colitis or crohn’s or Kidney dis* or urol* or nephr* or renal dis* or liver disease or hepatitis or HIV or AIDS or frailty or Neurocogn* or neurol* or dementia or amnestic or Alzheim* or Parkin* or epilepsy) AND (Depress* or Anxiety or Bipolar, Mania or Schizophr*, Schizoaff* or Psychot*, Psychos* or Obsess*, Neurosis or Post-traumatic* or PTSD or Trauma or Stress or Substance* or addict* or compuls* drug abuse or drug dependence or Disrupt*, Impulse* or Conduct).

Chinese Language

Chinese Language Academic Databases	Chinese Language Search Terms
• China National Knowledge Infrastructure (CNKI)<br>• Wanfang Data<br>• Airiti Library	Search terms related to COVID-19, mental health issues, and comorbid physical conditions: 新型冠状肺炎 (COVID-19) AND (心理健康 (mental health) AND 慢性病 (chronic illness) or 肿瘤 (tumour) or 糖尿病 (diabetes) or 心脏病 **(heart**disease) or 哮喘 (asthma) or 神经 (nerve) or 关节炎 (arthritis) or 肠病 (enteropathy) or 肾病 (kidney disease) or 艾滋病 (AIDS) or HIV (HIV) or 衰弱症 (frailty) or 肥胖症 (obesity) or 基础疾病 (underlying disease) or 既往疾病 (past disease)).

Table S2: English Literature Data Extraction Tables (n=60)

Citation and Country	Physical / Mental Health	Aim /Intervention/ Finding(s)	Study Design	Quality[a]
**Systematic Review, Meta-Analysis and Literature Reviews**				
1. Ceravolo MG et al. Systematic rapid living review on rehabilitation needs due to COVID-19: update to March 31st 2020. European Journal of Physical and Rehabilitation Medicine. 2020. **Italy**	Frail or frailty / Depression	**Aim:** Provide updates on the rehabilitation needs due to COVID-19. **Findings:** Rehabilitation interventions are described (e.g., music therapy, group rehabilitation), acute care, inpatient, and home interventions. It is recommended: 1) Early rehabilitation should be granted to inpatients with COVID-19; 2) people with restricted mobility due to quarantine or lockdown should receive exercise programs to reduce the risk of frailty, sarcopenia, cognitive decline, and depression; 3) tele-rehabilitation may represent the first option for people at home.	Systematic review	100%; Review based on 9 articles of varying methods
**RCTs**				
2. Wei N et al. Efficacy of internet-based integrated intervention on depression and anxiety symptoms in patients with COVID-19. Journal of Zhejiang University. Science. B. 2020, 1. **China**	HIV, CVD, hypertension, liver disease, gastric ulcers/ Anxiety, depression	**Aim:** A randomized, controlled, 2-week study of 26 COVID-19 patients (18-65 yrs) in an isolation ward screened for psychological distress (Patient Health Questionnaire-9 (PHQ-9); Generalized Anxiety Disorder-7 (GAD-7)). Exclusion: 1) PHQ-9 or GAD-7 of ⩾15; 2) suicidal ideation; 3) antipsychotic use; 4) inability to complete the assessment or follow instructions of the intervention. Groups: 1) internet-based integrated intervention (self-help intervention of breath relaxation training, mindfulness (body scan), "refuge" skills, and butterfly hug method) or supportive care (control group), with 13/group. The 17-item Hamilton Depression Scale (17-HAMD) and Hamilton Anxiety Scale (HAMA) assessed severity of depression and anxiety symptoms. **Findings:** 34.6% had underlying diseases: four had hypertension, two had liver disease and one each had gastric ulcer, coronary heart disease and AIDS. No significant difference between the two groups was observed at baseline for age, gender, illness severity, and PHQ-9, GAD-7, 17-HAMD, or HAMA scores. Significant reductions in mean scores of 17-HAMD ($F=37.35$, $P<0.001$) and HAMA ($F=26.58$, $P<0.001$), as well as a main effect of group (17-HAMD: $F=4.384$, $P=0.047$; HAMA:	RCT	80%; Small sample; potential bias related to unblinding; short follow-up

Citation and Country	Physical / Mental Health	Aim /Intervention/ Finding(s)	Study Design	Quality[a]
		F=5.634, P=0.026) and a group-by-time interaction (17-HAMD: F=5.268, P=0.009; HAMA: F=3.743, P=0.031) were reported. There were no main or interactive effects of age, gender, or severity of illness. Post hoc analyses by time points showed that scores of 17-HAMD and HAMA were significantly decreased in the intervention group at the end of the first (17-HAMD, t=−2.38, P=0.026; HAMA, t=−2.26, P=0.033) and second (17-HAMD, t=−3.09, P=0.005; HAMA, t=−3.75, P=0.001) weeks, when compared with the control group.		
**Case-Control/Retrospective Studies/Mixed Methods/Cross-Sectional**				
3. Shalash A et al. Mental health, physical activity and quality of life in Parkinson's disease during COVID-19 pandemic. Movement Disorders. 2020. **Egypt**	Parkinson's Disease (PD)/ Anxiety	**Aim:** Investigate the impact of COVID-19 pandemic on mental health, physical activities and quality of life (QoL) of patients with PD (n=38) and age/sex matched controls (n=20). Assessed using Depression, Anxiety, and Stress Scale–21 (DASS-21), Beck Depression Inventory, the short form of the international physical activity questionnaire (IPAQ) and the PD questionnaire (PDQ39). **Findings:** Most reported a negative impact on their mental health, physical activity, and health care and an interest in virtual visits. DASS ‐ total, depression, and anxiety were correlated with pre ‐ lockdown motor severity. DASS depression was positively correlated with pre ‐ lockdown; Beck Depression Inventory (BDI) and negatively with cognition. Total IPAQ scores were negatively correlated with total DASS (rs = −0.354, P = 0.029), DASS depression (rs = −0.441, P = 0.006), pre-lockdown motor severity–on, and BDI, but positively correlated with education and cognition. Total PDQ39 scores were significantly correlated with total and subscores of DASS, pre ‐ lockdown motor severity, BDI, and worry about unavailability of medication (rs = 0.347, P = 0.035). Compared with controls, patients showed significantly worse stress, depression, anxiety, total DASS, moderate physical activity, walking, total IPAQ, total	Cross-sectional/ comparisons	80%; Modest sample that tended to be higher educated

Citation and Country	Physical / Mental Health	Aim /Intervention/ Finding(s)	Study Design	Quality[a]
		and most of the PDQ39 dimensions PD patients showed a significant decline in physical activity compared with pre - lockdown (P = 0.002).		
4. Guo Q et al. Immediate psychological distress in quarantined patients with COVID-19 and its association with peripheral inflammation: A mixed-method study. Brain, Behavior, and Immunity. 2020, 88, 17-27. https://doi.org/10.1016/j.bbi.2020.05.038 **China**	Peripheral inflammation/ Depression, anxiety, PTS symptoms	**Aim:** Investigate the mental status of patients (n=103) with COVID-19 and age, education, gender, and place of residence matched controls (n=103); peripheral inflammatory markers also assessed. Excluded were those with lymphatic disorders or malignant hematologic diseases. Qualitative data were collected among five patients with COVID-19. **Findings:** Depression and anxiety symptoms were more common among COVID-19 patients than in normal controls. Those with COVID-19 had higher depression (P < 0.001), anxiety (P < 0.001), and post-traumatic stress symptoms (P < 0.001). Gender effect was observed in the score of "Perceived Helplessness", subscale of PSS-10, among female patients was higher (Z = 2.56, P = 0.010). C-reactive protein (CRP) correlated positively with the PHQ-9 total score (R = 0.37, P = 0.003) of those with depression. Change of CRP level from baseline inversely correlated with the PHQ-9 total score (R = -0.31, P = 0.002). Qualitative analysis revealed reports of negative feelings, including fear, guilt, and helplessness with stigma and uncertainty of viral disease progression as main concerns expressed by COVID-19 patients.	Mixed-methods approach – survey and interview of subsample	80%; Modest sample; authors indicate may not represent those with less severe COVID-19 symptoms; not all confounders ruled out; social desirability bias
5. Vanni G et al. Breast cancer and COVID-19: The effect of fear on patients' decision-making process. In Vivo. 2020, 34(3 suppl):1651-9. **Italy**	Cancer, breast (BC)/Anxiety	**Aim:** Assess the effect of BC patients' anxiety caused by the fear of COVID-19 on their decision- making process regarding treatment. **Finding:** Eighty-two patients with a suspected breast lesion (SBL) were divided into two groups: PRE- and POST-COVID-19-SBL. Seventy-eight breast cancer (BC) patients were divided into PRE- and POST-COVID-19-BC. Significant difference was found in the procedure refusal (PR) rate between the PRE- and POST-COVID-19 periods (p=0.0065), 9.30% vs 35.90%. Surgical refusal (SR) distribution demonstrated significant difference between PRE-COVID-19-BC and POST-COVID-19-BC groups	Retrospective descriptive	60%; Potential confounders / limited sample/ potential selection bias/ short period of

Citation and Country		Physical / Mental Health	Aim /Intervention/ Finding(s)	Study Design	Quality[a]
			(p=0.0208). POST-COVID group: 7 patients specified COVID-19 in the written refusal.		analysis (~2 months)
6.	Slaunwhite AK et al. Overdose and risk factors for coronavirus disease 2019. Drug and alcohol dependence. 2020, 212, 108047. **Canada**	Respiratory, COPD/ Substance use disorders	**Aim:** Quantify the prevalence of risk factors associated with mortality due to COVID-19 for persons who have non-fatal overdose between 2015-17 compared with those who do not have such an overdose. **Findings:** Previous non-fatal overdose increased the odds of having the comorbid conditions investigated: chronic respiratory disease (OR 2.01, 95% CI 1.92-2.11, p<0.001) diabetes (OR 1.24, 95% CI 1.16-1.32), coronary artery disease (OR 2.08, 95% CI 1.92-2.27. p<0.001), and Elixhauser Comorbidity Index > 2 (OR 18.05, 95% CI 17.19-18.96, p<0.001). There was no significant difference in the prevalence of hypertension among those who overdosed and those who didn't.	Retrospective analysis of administrative data	80%; Likely under-reporting of overdose due to mis-classification bias
7.	Lara B et al. Neuropsychiatric symptoms and quality of life in Spanish patients with Alzheimer's disease during the COVID-19 lockdown. Eur J Neurol. 2020. **Spain**	Alzheimer’s Dementia, Mild Cognitive Impairment/ Anxiety, neuropsychiatric symptoms	**Aim:** Analyse Alzheimer's (AD) and Mild Cognitive Impairment (MCI) patients' neuropsychiatric symptoms and quality of life due to the impact of the pandemic lockdown. The sample size was 20 AD patients and caregivers and 20 MCI patients and caregivers. **Findings:** Reported worsening of neuropsychiatric symptoms in patients with AD and MCI during 5 weeks of lockdown, with agitation, apathy and aberrant motor activity being the most affected symptoms. Most frequently affected neuropsychiatric symptoms were apathy [4.15 (3.78) vs. 5.75 (4.02); p=0.002] and anxiety [3.95 (3.73) vs. 5.30 (4.01); p=0.006] in patients with MCI, and apathy [2.35 (2.70) vs. 3.75 (3.78); p=0.036], agitation [0.45 (1.14) vs. 1.50 (2.66); p=0.029] and aberrant motor behaviour [1.25 (2.86) vs. 2.00 (2.93); P = 0.044] in those with AD.	Cross-sectional	80%; Potential recall bias; modest sample

Citation and Country	Physical / Mental Health	Aim /Intervention/ Finding(s)	Study Design	Quality[a]
8. Pulvirenti F et al. Health-Related-Quality of Life in Common Variable Immunodeficiency Italian patients switched to remote assistance during the COVID-19 pandemic. The Journal of Allergy and Clinical Immunology: In Practice. 2020. **Italy**	Respiratory, other/Anxiety	**Aim:** Health-related quality-of-life assessments help to identify major issues and patients at risk of anxiety/depression in the coronavirus disease 2019 pandemic. **Finding:** The quality of life was surveyed in 158 patients with PADs by the Common Variable Immune Deficiency Quality of Life questionnaire, a disease-specific tool. The coronavirus disease 2019 epidemic impacted HRQOL and the risk of anxiety/depression of patients with PADs. The remote assistance program was a useful possibility to limit personal contacts without influencing the HRQOL.	Cross-sectional	60%; Modest sample; respondents may have been those most impacted
9. Naser MA. One Aspect of Coronavirus disease (COVID-19) Outbreak in Iran: High Anxiety among MS Patients. Multiple sclerosis and related disorders. 2020, 41, 102138. **Iran**	Multiple Sclerosis/ Anxiety	**Aim:** Measure anxiety levels amongst MS patients during the COVID-19 pandemic. **Findings:** The mean level of anxiety (Becker Anxiety Inventory Questionnaire) amongst participants was 25.72±6.53, which is within the range of moderate to severe.	Cross sectional	60%; Anxiety levels pre COVID-19 not mea-sured, limited sample, no control
10. Gómez-Salgado J et al. Related Health Factors of Psychological Distress During the COVID-19 Pandemic in Spain. International Journal of Environmental Research and Public Health. 2020, 17(11), 3947. **Spain**	Respiratory, other/Anxiety	**Aim:** Analyse psychological distress in Spain during the COVID-19 pandemic, identify role of socioeconomic factors, physical symptoms, and other variables. Hypothesized that COVID-19 does not generate psychological distress in the population. A final sample of 4180 people over the age of 18 analyzed. **Findings**: Physical health conditions reported included high blood pressure (29.0%), chronic respiratory disease (25.3%), diabetes (8.3%), immunosuppression disease or situation (7.0%), metabolic syndrome (5.9%), chronic cardiovascular disease (5.0%), and active cancer (2.6%). 72.0% of participants had some form of psychological distress (GHQ-12 score >8). Distressed	Cross-sectional	60%; Limited general-izability; non-probability sampling

Citation and Country		Physical / Mental Health	Aim /Intervention/ Finding(s)	Study Design	Quality[a]
			individuals were more often female (79.6% female vs 20.4% male, p<0.001) and significantly younger (mean age 39.03 vs 43.43, p<0.001) than non-distressed individuals. There was greater distress in those working outside the home (48.5% vs 18.8% working at home and 32.7% not working, p<0.001) and those not living with children <16 years old (50.3% vs 49.7%, p=0.007). Those who presented symptoms of COVID-19 had significantly higher psychological distress for all physical symptoms assessed (p<0.001 in all cases). This study describes risk factors for psychological distress during the COVID-19 pandemic using a cross-sectional design based on a representative sample of the Spanish population. Future health policy measures should be taken to direct resources to those in groups identified as high-risk of psychological distress		
11.	Ozamiz-Etxebarria N et al. Stress, anxiety, and depression levels in the initial stage of the COVID-19 outbreak in a population sample in the northern Spain. Cadernos de Saúde Pública. 2020, 36, e00054020. **Spain**	Chronic disease/ Anxiety, depression	**Aim:** Assess stress, anxiety, and depression with the arrival of the virus and the levels of symptoms according to age, comorbidity, and confinement in a population in northern Spain. Questionnaires were sent to a total of approximately 2,400 individuals, of whom 1,003 replied (recruited March 11-15, 2020, from the three provinces of the Basque Autonomous Community. **Findings:** Severe and extremely severe levels of stress, anxiety, and depression in the sample in the Basque Autonomous Community in Spain (at initial phase of outbreak) were lower than in the study in China; The Basque (Spain) sample showed higher mean levels of symptoms (stress, anxiety, and depression) after March 14, when the population began to feel the effects of the stay-at-home order; Individuals reporting chronic diseases had higher mean levels of stress, anxiety, and depression compared to participants not reporting such diseases; Unexpectedly, there were higher mean levels of stress, anxiety, and depression in the 18-25-year age bracket, followed by the 26-60-year bracket.	Cross-sectional exploratory-descriptive study	60%; Non-response bias; limited general-izability; unable to report between group statistical significance

Citation and Country	Physical / Mental Health	Aim /Intervention/ Finding(s)	Study Design	Quality[a]
12. Sidor A, Rzymski P. Dietary choices and habits during COVID-19 Lockdown: Experience from Poland. Nutrients. 2020, 12(6), 1657. **Poland**	Obesity/ Substance Use, stress	**Aim:** Assess whether nutritional and consumer habits have been affected during the nationwide COVID-19-related quarantine in Poland. Sample were 1097 Polish people age ≥ 18 years old, and female or male gender. Individuals working on a regular basis during the lockdown were excluded. **Findings:** 43.5% of surveyed individuals reported eating more during quarantine, and 51.8% admitted to snacking between meals more frequently. Increased food consumption and snacking was more prevalent in individuals with higher BMI; demonstrates that quarantine may pose a significant dietary risk, particularly for overweight and obese individuals. Almost 15% (14.6%) reported an increase in alcohol consumption; this was reported to be higher among those who indicated they had an alcohol addiction. Eating breakfast every day was more often seen in individuals with higher education (72.3%) than with vocational (66.7%), secondary (58.9%), and primary (42.9%) education level.	Cross-sectional online survey	60%; Modest sample; non-response bias; under-representa-tion of men noted
13. Sanchez TH et al. Characterizing the Impact of COVID-19 on men who have sex with men across the United States in April, 2020. AIDS and Behavior. 2020, 1-9. **USA**	HIV/Anxiety, substance use	**Aim:** Assess the COVID-19 related impacts on the sexual health of men who have sex with men (MSM). **Findings:** Sample consisted of 1052 US men who have sex with men (MSM) When asked about compliance with local COVID-19 measures, 20.1% reported 100% adherence, 71.4% reported greater than 50% but less than 100% adherence, and 8.5% reported less than 50% adherence to the measures (n = 190 did not answer); Majority of participants had decreased quality of life, increased anxiety (73.4%), and decreased connection to friends due to COVID-19; Large proportions of participants had also experienced problems with basic resource needs (e.g., difficulty buying food, paying rent) - more likely in younger participants; 19.1% of participants reported losing their job due to COVID-19; increased use of recreational drugs and increased alcohol consumption; Many participants reported problems accessing HIV or STI testing and STI treatment due to COVID-19 or the plans to manage it (more	Cross-sectional online survey	60%; Social desirability noted by authors; no comparison group

Citation and Country		Physical / Mental Health	Aim /Intervention/ Finding(s)	Study Design	Quality[a]
			prevalent in younger participants); 97.9% of participants were willing to collect specimens at home for mailed-in SARS-CoV-2 COVID-19 testing for diagnostic purposes.		
14.	Umucu, E., Lee, B. Examining the impact of COVID-19 on stress and coping strategies in individuals with disabilities and chronic conditions. Rehabilitation Psychology. 2020, *65*(3), 193–198.<br>**USA**	Co- and Multi-morbidities/ Depression, anxiety, stress	**Aim:** Describe the perceived stress levels and coping mechanism related to COVID-19 in individuals with chronic conditions or disabilities. **Finding**: Participants reported moderate levels of stress, anxiety, and depression. The most common coping strategies among the participants were acceptance and self-distraction. Perceived stress was positively correlated with the coping strategies of self-distraction, substance abuse, venting, and behavioural disengagement. The coping strategies positively associated with well-being were emotional support, humour, and religion. In a global pandemic situation, individuals with chronic health conditions and disabilities are disproportionately affected. Understanding this impact can lead to the development of improved care and support for these individuals	Cross-sectional online survey	60%; Convenience sample; Primarily White, educated, English-speaking.<br>Some sub-scales of Brief-COPE have low reliability; no strati-fication by condition
15.	Germani A et al. Emerging Adults and COVID-19: The Role of Individualism-Collectivism on Perceived Risks and Psychological Maladjustment. International Journal of Environmental Research and Public Health. 2020, 17(10), 3497.<br>**Italy**	History of chronic physical diseases/ Anxiety, stress, history of psychological disorders	**Aim:** Evaluate **a)** emerging adults' perceived knowledge and worries about COVID-19; **b)** emotional and behavioral difficulties, state anxiety, and stress during lockdown; **c)** association between (a) and (b); **d)** relationships between cultural orientations at the individual level and the aforementioned aspects; **e)** role of cultural dimensions on psychological maladjustment. **Finding:** 1101 participants; 31.9% with history of psychological conditions and 6.5% with history of a chronic physical condition. Reported high mean scores as to perceived knowledge, worries, and concerns about COVID-19; STAI-Y State Scale shows presence of relevant levels of anxiety related to the	Cross-sectional online survey;	60%; Non-probability sample; no specific information by health condition

Citation and Country	Physical / Mental Health	Aim /Intervention/ Finding(s)	Study Design	Quality[a]
		specific moment, and PSS total score fall into the range of moderate stress; knowledge, as well as worries and concerns about COVID-19, were significantly and positively related to psychological maladjustment in terms of emotional/ behavioral difficulties, state anxiety, and stress; Only horizontal collectivism was significantly and negatively correlated to emotional/behavioral difficulties, state anxiety, and stress.		
16. Wang C et al. Immediate Psychological Responses and Associated Factors during the Initial Stage of the 2019 Coronavirus (COVID-19) Epidemic among the General Population in China. International Journal of Environmental Research and Public Health. 2020 **China**	Self-reported health and history of chronic illness/ Anxiety, depression	**Aim:** Survey the general public to better understand their levels of psychological impact, anxiety, depression, and stress during the initial stage of the COVID-19 outbreak. This study included 1210 respondents from 194 cities in China. **Findings:** Female gender, student status, and specific physical symptoms were associated with a greater psychological impact of the outbreak and higher levels of stress, anxiety, and depression. Poor or very poor self-rated health status was significantly associated with a greater psychological impact of the outbreak (B=0.76, 95% CI 0.02-1.49), and higher DASS stress subscale (B=0.45, 95% CI 0.02-0.88), DASS anxiety subscale (B=0.90, 95% CI 0.22-1.58), and DASS depression subscale (B=0.65, 95% CI 0.10-1.20) scores as compared to those with very good or good self-rated health status. History of chronic illness was significantly associated with higher IES-R, DASS stress subscale, DASS anxiety subscale, and DASS depression subscale scores.	Cross-sectional questionnaire	60%; General discussion about physical health; no specific details by condition; limited general-izability

Citation and Country	Physical / Mental Health	Aim /Intervention/ Finding(s)	Study Design	Quality[a]
17. Nachimuthu S et al. Coping with diabetes during the COVID–19 lockdown in India: Results of an online pilot survey. Diabetes & Metabolic Syndrome: Clinical Research & Reviews. 2020 **India**	Diabetes (type 1/2)/ Anxiety, stress	**Aim:** An online-based pilot survey was conducted to study how people with diabetes were coping with their diabetes during COVID-19 lockdown. **Findings:** People with diabetes, especially those on multiple OAD and insulin, should monitor their blood sugar levels more often. The sample size was a total of 100 patients participated in the survey. 92% of the participants had Type 2 diabetes and 54% were males. Majority of the participants were from the age group of 65 or more. Most of the participants had diabetes for more than 5 years. 65% of the study participants had a duration of diabetes from 5 to 10 years.	Cross-sectional Survey	60%; Modest sample, convenience sample
18. Salari M et al. Incidence of Anxiety in Parkinson's Disease During the Coronavirus Disease (COVID-19) Pandemic. Movement Disorders. 2020. **Iran**	Parkinson's disease/ Anxiety	**Aim:** Provided a survey that evaluated level of anxiety among those affected by Parkinson's Disease (PD). The sample size was 137 PD patients, 95 PD caregivers and 442 control (random population). **Findings:** Higher rates of anxiety (BAI-II; Persian score) was reported in patients / caregivers surrounding PD. Main concern was surrounding drug availability in lockdown. Main concern of PD patients / caregivers surrounded drug availability during quarantine. Could look into ways to mitigate this. A high proportion of sample had pre-existing medical conditions (e.g., cardiac disease, obesity, diabetes).	Cross-sectional survey	60%; Modest and non-probability sample; multi-morbidities in many respondents
19. Zipprich HM et al. Knowledge, Attitudes, Practices, and Burden During the COVID-19 Pandemic in People with Parkinson's Disease in Germany. Journal of Clinical Medicine. 2020, 9(6), 1643. **Germany**	Parkinson's disease/ Anxiety, stress	**Aim:** Examine the adherence to preventive measures for COVID-19 in Parkinson's Disease Patients through looking at the Knowledge, Attitudes and Critical Application of the patients towards COVID-19. Authors conducted semi-structured telephone interviews were performed with 99 patients with Parkinson's disease (PD) and 21 controls to explore knowledge, attitudes, practices, and burden in order to elucidate nonadherence to preventive measures. Information from their medical records was extracted including demographics, information about cognitive state (Montreal Cognitive Assessment (MoCa)), depressive mood (Beck's Depression Inventory II (BDI)), motor function (Movement Disorder Society–	Cross-sectional survey	60%; Modest non-probability sample; relied on medical chart data

Citation and Country	Physical / Mental Health	Aim /Intervention/ Finding(s)	Study Design	Quality[a]
		sponsored revision of the Unified Parkinson's Disease Rating Scale III (MDS-UPDRS III)), presence of non-motor symptoms (Revised Non-motor Symptoms Questionnaire (NMS-Q)) , and adherence to medication (Stendal Adherence with Medication Score; SAMS). Data on these clinical parameters were obtained from two to six months before the interviews were performed. **Findings:** Majority of patients understood the preventive measures and felt sufficiently informed. Analysis of qualitative answers, however, showed that about 30% of patients had an insufficient level of knowledge, which was not associated with educational level, cognitive disorders, or depression.		
20. Brown TS et al. The Effect of The COVID-19 Pandemic On Electively Scheduled HIP and KNEE Arthroplasty Patients in THE United States. The Journal of Arthroplasty. 2020 **USA**	Arthritis, osteoarthritis/ Anxiety	**Aim: I**dentify hip and knee arthroplasty patients in the US (n=360) who had their scheduled procedure postponed/canceled because of the COVID-19 pandemic and assess their pain, anxiety, physical function, and economic ability to undergo a delayed operation once the threat of COVID-19 has subsided. **Findings**: Not knowing when the procedure will be rescheduled was the highest source of anxiety; Not knowing when the procedure will be rescheduled caused moderate or severe anxiety in 217 patients (60%); Becoming infected with COVID-19 and spreading infection to others were also high causes for anxiety; AGE: For the younger cohort, the questions about finances and job insecurity caused significantly more anxiety than in the older group; **G**EOGRAPHY**:** In the Northeast, patients had significantly higher anxiety around becoming infected with COVID-19; PAIN**:** Most patients stated that their pain from hip/knee arthritis has increased since surgery cancellation (54%), and 30% stated that they would have proceeded with surgery in spite of the risks posed by COVID-19; Public health: Most patients (85%) agreed with the decision to stop elective procedures in the face of ongoing pandemic.	Cross-sectional survey	60%; Modest sample; reporting bias noted

Citation and Country	Physical / Mental Health	Aim /Intervention/ Finding(s)	Study Design	Quality[a]
21. Rodler S et al. Telehealth in Uro-oncology Beyond the Pandemic: Toll or Lifesaver? European Urology Focus, 2020, 6(5), 1097–1103. **Germany**	Genitourinary cancers/ Anxiety	**Aim:** Determine the perspective of oncology patients on the use of telehealth during the pandemic. The sample consisted of 101 Genitourinary cancer patients undergoing treatment at a uro-oncology unit. **Findings:** For most patients, their anxiety over cancer superseded that of COVID-19. A majority of patients also opposed interruptions to their treatment. The rating for telehealth during the current crisis was high, but the rating for maintaining telehealth beyond the pandemic was low. Most patients rated their susceptibility to COVID-19 as being equal to or lower than that of the general population. The findings from this study could inform the future of telehealth and treatment options for cancer patients.	Cross-sectional survey	60%; Modest sample and non-probability sampling
22. Rozanova et al. (2020). Social Support is Key to Retention in Care during COVID-19 Pandemic among Older People with HIV and Substance Use Disorders in Ukraine. Substance Use & Misuse, 2020, 55(11), 1902–1904. **Ukraine**	HIV/Substance use disorder	**Aim:** Examine COVID-19 pandemic impacts older patients with HIV and substance use disorders and their ability to continue HIV and/or addiction treatment. **Findings:** While older people with HIV and SUD maintained HIV and SUD therapy throughout COVID-19 lockdown, there is anxiety about the availability of treatment services. Providers were concerned about the stability of clinical services. Social support is critical to avoiding treatment interruption for older patients with HIV and SUD. A quarter of the participants reported having fewer than 2 people including healthcare providers who can support them, and 61% of responders had no treatment supporter.	Cross-sectional telephone survey	60%; Modest sample; convenience sample
23. Goodman-Casanova et al. (2020). Telehealth Home Support During COVID-19 Confinement for Community-Dwelling Older Adults With Mild Cognitive Impairment or Mild Dementia: Survey Study. Journal of Medical Internet	Dementia, Alzheimer's /Anxiety, Stress	**Aim: S**tudy the impact of confinement on the health and well-being of older adults with mild cognitive impairment/dementia living in community dwellings, while also providing television-based health and social support. A telephone based survey was administered to 93 participants (intervention and control) who had already been enrolled in the TV-AssistDem clinical trial from March 25- April 6, 2020. **Findings:** TV-AssistDem (TeleVision-based ASSistive Integrated Service to supporT European adults living with mild DEMentia or mild cognitive impairment) trial.	Cross-sectional telephone survey	60%; Mental health measure self-report of non-validated measures

Citation and Country	Physical / Mental Health	Aim /Intervention/ Finding(s)	Study Design	Quality[a]
Research, 22(5), e19434. **Spain**		The mental health and well-being (bored, sad, worried, afraid, anxious) and sleep quality of the majority of the participants was optimal during the COVID-19 lockdown and no differences were noted between the intervention and control. Those living alone experienced greater negative psychological effects and sleeping problems. TV sets were the preferred technological device to obtain COVID-19 information, and those with the TV-AssistDem trial demonstrated greater potential for cognitive stimulation. This study provides insight on the development of interventions that can be used to provide support to older adults during the COVID-19 pandemic.		
24. Walędziak M et al. Bariatric Surgery during COVID-19 Pandemic from Patients' Point of View—The Results of a National Survey. Journal of Clinical Medicine. 2020, 9(6), 1697. **Poland**	Obesity, diabetes (type 2)/ Anxiety	**Aim:** Investigate the impact of the COVID-19 pandemic on bariatric care from the patients' point of view. The sample consisted of 800 bariatric patients who completed the online survey were included in the study (no mention of any exclusions). **Findings:** 64% of respondents had contact with patients with confirmed COVID-19 or were staying in quarantine; 21.9% of patients were treated in bariatric centres that currently manage COVID-19 patients; The majority (74.53%) of patients felt more anxiety/fear about their health in regard to the present epidemiologic state; In 172 cases (69.36%), the date of bariatric surgery was postponed due to the COVID-19 pandemic; Regardless of the risk of becoming infected with COVID-19, 42.69% of patients would like to have a visit in a bariatric clinic. Many patients believed that bariatric procedures should be resumed when COVID-19 cases start to decrease, WHO declares the end of the pandemic, all COVID-19 patients are discharged, or a vaccine is introduced; The majority of responders were aware of the fact that obesity was an important risk factor that could impair the course of COVID-19 disease.	National survey (cross-sectional)	60%; Modest sample; recall and social desirability bias noted by authors

Citation and Country	Physical / Mental Health	Aim /Intervention/ Finding(s)	Study Design	Quality[a]
25. Hao X et al. Severe psychological distress among patients with epilepsy during the COVID-19 outbreak in southwest China. Epilepsia. 2020 **China**	Epilepsy/ Anxiety	**Aim:** Compare the severity of psychological distress among epileptic patients in China (n=252) and controls (n=252). **Findings:** Diagnosis of drug-resistant epilepsy and amount of time spent daily on media coverage of COVID-19 were associated with increased prevalence of severe psychological distress. Overall, there was a significantly greater level of psychological distress (Kessler Psychological Distress Scale – 6 item) in inpatients than controls during COVID-19	Web-based, self-report survey. Cross-sectional	80%; Consecutive sample / clinical population
26. Meng H et al. (2020). Analyze the psychological impact of COVID-19 among the elderly population in China and make corresponding suggestions. Psychiatry Research, 289, 112983. **China**	Reference to “debilitated physical quality” and chronic medical history /Anxiety, Depression	**Aim:** Survey older adults about psychological status during the period of “COVID-19. **Findings:** The questionnaire included questions about demographics, chronic medical history, previous mental illness, whether there are confirmed Coronavirus cases around them, whether to receive trainings on pandemic related knowledge, PHQ-9 Questionnaire, and GAD-7 Questionnaire. GAD-7 and PHQ-9. Findings: A total of 1556 of which 954 were female and most were 60 to 64 years, 37.1% experienced depression and anxiety. There were gender differences in emotional response, with women experiencing more anxiety and depression than men. No significant differences in mental health by age. Based on the results, psychological intervention strategies for were suggested that included: 1) Concentrate psychological attention on female seniors, low educated seniors, divorced and widowed seniors, and those who are living alone, having sleep problems and mental health issues; 2) improve systems for psychological counseling and psychological crisis intervention for the seniors, and psychological health education in the media; 3) where emotional response is serious, call a psychological hotline, online consultation, or visit a professional institution.	Cross-sectional survey	80%; Non-probability sample; self-report measures of physical and mental health
**Consensus Statements**				
27. Barker-Davies RM et al. The Stanford Hall consensus	Respiratory, bronchitis	**Aim:** Provide an overarching series of recommendations by assimilating the current evidence base for, and likely	Consensus/ clinical	AGREE II

Citation and Country	Physical / Mental Health	Aim /Intervention/ Finding(s)	Study Design	Quality[a]
statement for post-COVID-19 rehabilitation. British Journal of Sports Medicine. 2020 **United Kingdom**	(primary), CVD / Anxiety, Depression	requirements of, rehabilitation after COVID-19. The intended patient target is an active population, including military personnel and athletes with the desire to optimise recovery and human performance in occupational settings. **Findings:** A series of clinical guidelines for rehabilitation are provided for various systems including MSK, cardiac, pulmonary. The study also describes several pulmonary, cardiac, and psychological rehabilitation programs. PR programmes can be delivered within a hospital setting, outpatient, home-based or even remotely supervised, with the majority in the UK outpatient based. If cardiac pathology is present, specific cardiac rehabilitation programmes should be provided tailored to the individual based on their cardiac complications, impairments and rehabilitation needs assessment.	Recommend-dations	60%; Recall bias; generalizabil-ity
28. Algarin AB et al. Symptoms, Stress, and HIV-Related Care Among Older People Living with HIV During the COVID-19 Pandemic, Miami, Florida. AIDS and Behavior. 2020, doi:10.1007/s10461-020-02869-3 **USA**	HIV/ Stress	**Aim:** Determine the impact of COVID-19 on the patient care and mental health of people living with HIV (PWLH). **Findings**: Stress levels in PWLH were impacted due to COVID-19, and that weekly phone calls can allow patients to stay connected while also offering them the opportunity to receive any necessary assistance. New guidelines should be developed for the care of persons with HIV regarding telehealth methods, mental health assessments, and patient education. The areas suggested in this study could be targeted for future PWLH patient guidelines, care, and education.	Consensus/ clinical recommend-dations	60%; 5/24 lost to follow up, and 3 other enrolled participants were not reached during the first call, small sample
**General Reviews/Commentaries, Case Studies, and Opinion Pieces**				
29. S. Cuschieri. Grech S. COVID-19 and diabetes: The why, the what and the how, Journal of Diabetes and Its Complications, 2020, 34 (9), **China**	Type 2 Diabetes (primary), obesity/ Psychological Stress, Anxiety and Depression	**Aim:** Summarize information and resources available on diabetes and COVID-19 and special measures that individuals with diabetes need to follow. **Findings:** COVID-19 may enhance complications in individuals with diabetes through an imbalance in angiotensin-converting enzyme 2 (ACE2) activation pathways leading to an inflammatory response. These individuals may be	Literature review	N/A; Lack of clinical trials and other systematic reviews in search

Citation and Country	Physical / Mental Health	Aim /Intervention/ Finding(s)	Study Design	Quality[a]
		prone to worsened COVID-19 complications- general preventive measures, remaining hydrated, monitoring blood glucose regularly and monitoring ketone bodies in urine if on insulin is essential. All this while concurrently maintaining physical activity and a healthy diet. The study suggests preventative measures and recommendation for people with diabetes during the COVID-19 pandemic.		
30. Brown E et al. Anticipating and Mitigating the Impact of the COVID-19 Pandemic on Alzheimer's Disease and Related Dementias. The American Journal of Geriatric Psychiatry, 2020, 28(7), 712-721. **Canada**	Dementia, Alzheimer's, co- and multi-morbidities/ Anxiety, depression	**Aim**: Highlight mitigation strategies for patients with Alzheimer's Disease and related dementias (ADRD) in relation to the COVID-19 pandemic. **Findings:** ADRD patients are at a higher risk due to their age and comorbidities. Additionally, COVID-19 has impacted the ability of patients to receive care and treatment. Isolation inhibits patients from reaching out to their social support network, thereby increasing fear and anxiety amongst them. Incorporating alternative social/physical activities within homes, using technology to stay connected and receive certain healthcare services, making contingency plans. The proposed recommendations could be incorporated into future policies in order to mitigate the impact of COVID-19 on ADRD patients.	Review	N/A

Citation and Country	Physical / Mental Health	Aim /Intervention/ Finding(s)	Study Design	Quality[a]
31. Rogers BG et al. Adapting Substance Use Treatment for HIV Affected Communities During COVID-19: Comparisons Between a Sexually Transmitted Infections (STI) Clinic and a Local Community Based Organization. AIDS and Behavior. 2020, 1. **USA**	HIV/Substance use	**Aim:** Describe changes in response to COVID-19 made to a substance use treatment program people at risk or living with HIV, primarily for men who have sex with men who use stimulants and/or opioids. **Findings:** Most psychotherapy services were transitioned to electronic means following APA guidelines. Telemedicine: Clinical psychologists were trained in telemedicine, remote therapy guidelines were developed, and appropriately secure software was procured. This transition decreased access to care to those who did not have electronic means - none of the clients previously treated at the community-based site (rather than the STI site) continued to receive treatment. While in person psychotherapy ceased, the community-based site continued to offer basic needs and harm reduction supplies. Less formal support ("peer-recovery") was administered through Facebook, text, or in person as it is not subject to the same strict privacy regulations as telemedicine.	Case example of a health program	N/A
32. Pope JE. What does the COVID-19 pandemic mean for rheumatology patients?. Current Treatment Options in Rheumatology. 2020, 1. **Canada**	Arthritis, rheumatoid/ Anxiety	**Aim:** Discuss how the COVID-19 pandemic affects people with autoimmune rheumatic disease. **Findings:** Patients with rheumatoid arthritis are not more prone to develop significant health complications during COVID-19 infection. Shortages of Hydroxychloroquine may cause anxiety for individuals suffering from with rheumatic diseases. Medications for treating rheumatoid arthritis should be carefully distributed. A lack of regulation can lead to negative repercussions for individuals suffering from the disease	Narrative review	N/A
33. Chevance A et al. Ensuring mental health care during the SARS-CoV-2 epidemic in France: a narrative review. L'encephale. 2020 . **France**	CVD, other, obesity, diabetes / Schizophrenia spectrum, depression	**Aim:** Show that the major difficulties in coordinating and managing this crisis highlight the absolute necessity of a common voice for French psychiatry with the health authorities. **Findings:** Identified four types of major vulnerabilities among patients with mental disorders during this pandemic: (1) medical comorbidities that are more frequently found among patients with mental disorders (cardiovascular and pulmonary	Narrative review	N/A

Citation and Country	Physical / Mental Health	Aim /Intervention/ Finding(s)	Study Design	Quality[a]
		pathologies, diabetes, obesity, etc.) which are risk factors for severe COVID-19 infection; (2) age (the elderly form the population most vulnerable to the coronavirus); (3) cognitive and behavioural disorders, which can hamper compliance with confinement and hygiene measures and finally and (4) psychosocial vulnerability as a result of stigmatization and/or socioeconomic difficulties. Furthermore, the mental health healthcare system is more vulnerable than other healthcare systems		
34. Condie LO. Neurotropic mechanisms in COVID-19 and their potential influence on neuropsychological outcomes in children. Child Neuropsychology. 2020, 26:5, 577-596. **United States**	Cognitive impairment/ Anxiety, depression	**Aim:** Provide recommendations for some initial updates in neuropsychology practice during the COVID-19 pandemic in children. **Findings:** There are COVID-19 implications for neuropsychological functioning that include changes in cognitive, motor, and language abilities, executive functioning impact, medical coping difficulties, psychosocial impact, and independent functioning impact.	Narrative review	N/A
35. Mehra A et al. A crisis for elderly with mental disorders: Relapse of symptoms due to heightened anxiety due to COVID-19. Asian Journal of Psychiatry. 2020, 51, 102114. **India**	CVD, hypertension, Diabetes mellitus/ Depression (primary), anxiety	**Aim:** Raise awareness about mental health issues in the elderly population during the COVID-19 pandemic. Two case examples (one male/one female) were presented of individuals admitted to hospital with relapse of depressive disorder. **Findings:** COVID-19 can lead to heightened levels of anxiety in elderly patients. It is important to provide elderly patients with therapies to help them deal with mental health issues during the pandemic. The interventions included psychotherapy, escitalopram, clonazepam, and olanzapine. The authors highlighted that part of prevention of mental health issues in the context of the pandemic that fears about the elderly be minimized. An example included avoiding words on social media like "#BoomerRemover". Both cases were preoccupied with thoughts of being vulnerable to get the infections and dying to the COVID-	Case studies (2 cases presented)	N/A

Citation and Country	Physical / Mental Health	Aim /Intervention/ Finding(s)	Study Design	Quality[a]
		19. The report recommends a shift in media practices to decrease anxiety levels in patients		
36. Armbruster M et al. Addressing Health Inequities Exacerbated by COVID-19 Among Youth With HIV: Expanding Our Toolkit. Journal of Adolescent Health. 2020. **United States**	HIV/ Anxiety, Bipolar, Schizophrenia, ADHD, Autism, Intellectual delay, substance use	**Aim:** Chronicle the structural and logistic approaches that a team undertook to proactively address the social determinants of health that will be negatively impacted by the COVID-19 pandemic, while supporting youth with HIV (YHIV) to maintain medication adherence and viral suppression. **Findings:** It was anticipated that during and after the pandemic, these challenges would increase their risk of COVID-19 exposure and negatively impact treatment engagement, medication adherence, and viral suppression, resulting in disease progression and increased HIV transmission. A toolkit was developed with various actions and resources to address the health needs of youth with HIV and COVID-19. This program could have wider policy implications if implemented on a larger scale.	Case study of a health program	N/A
37. Marsden J et al. Mitigating and learning from the impact of COVID-19 infection on addictive disorders. Addiction. 2020 **United Kingdom**	Cardiovascular diseases/ Substance use (opioid overdose)	**Aim:** Summarize threats of the COVID-19 pandemic to people with addictive disorders and suggest methods of addressing those issues. **Findings:** Opioids: Social distancing measures will likely reduce the ability of people with opioid use disorder to access healthcare. The UK encouraged healthcare providers to give 14-day supply of opioid use disorder medications for self-administration rather than supervised consumption at a safe injection site, which may increase fatal opioid related overdoses. Stimulants: Stimulant use increases risk of cardiovascular disease, a major risk factor for COVID-19 mortality. The effects of all these substances will likely most affect those living in poverty and with additional mental health disorders.	Editorial	N/A
38. Khalatbari-Soltani et al. Importance of collecting data on socioeconomic determinants from the early stage of the COVID-19	Authors identify physical health conditions that	**Aim:** Identify how socioeconomic position and other social factors relate to the epidemiology of COVID-19. **Findings:** Socioeconomic positions such as low income, labour-based occupations and lower educational levels are likely to be	Review	N/A

Citation and Country	Physical / Mental Health	Aim /Intervention/ Finding(s)	Study Design	Quality[a]
outbreak onwards. J Epidemiol Community Health, doi: 10.1136/ jech-2020-214297 **Australia**	increase risk/ Anxiety	determinants of COVID-19 infection, severity and mortality. The authors highlighted barriers and issues among ethnic groups, by sex/gender, and geographical contexts. The authors highlight that socio-economic variables should be considered as clinical factors. It suggests that pandemic recommendations and guidelines. provided by international and national agencies need to recognise the collective contribution of the social determinants of health and their intersectionality to pandemic risk mitigation.		
39. Bikson M et al. Guidelines for TMS/tES clinical services and research through the COVID-19 pandemic. Brain Stimulation. 2020 **USA**	Depression (primary), anxiety/ stroke	**Aim:** Facilitate the re-establishment of access to non-invasive brain simulation (NIBS) clinical services and research operations during the current COVID-19 pandemic and possible future outbreaks, we develop and discuss a framework for balancing the importance of NIBS operations with safety considerations, while addressing the needs of all stakeholders. We focus on Transcranial Magnetic Stimulation (TMS) and low intensity transcranial Electrical Stimulation (tES) - including transcranial Direct Current Stimulation (tDCS) and transcranial Alternating Current Stimulation (tACS). **Findings:** There is an evident need to maintain NIBS operations through the pandemics and addressing effects of COVID-19 on brain and mind. A model based on three phases (early COVID-19 impact, current practices, and future preparation) with an 11-step checklist (spanning removing or streamlining in-person protocols, incorporating telemedicine, and addressing COVID-19-associated adverse events) is proposed. Recommendations on implementing social distancing and sterilization of NIBS related equipment, specific considerations of COVID-19 positive populations including mental health comorbidities. We discuss COVID-19 considerations specifically for stroke, addiction, and pediatrics.	Expert consensus paper based on survey to institutions applying NIBS	N/A

Citation and Country	Physical / Mental Health	Aim /Intervention/ Finding(s)	Study Design	Quality[a]
40. Dell NA et al. Service Needs of Older Adults with Serious Mental Illness. Journal of Gerontological Social Work. 2020, 1-3. **USA**	Schizophrenia spectrum, Bipolar and related disorders, Major depressive disorder, trauma exposure/PTSD	**Aim:** Inform social workers of psychosocial needs of older adults with serious mental illnesses during the COVID-19 pandemic as well as interventions to meet these needs. **Findings:** Addresses some interventions that may reduce the impact of social circumstances on the mental and physical health of older adults, although this was a separate study from the existing literature.	Letter to editor with reference to interventions	N/A
41. Padala SP et al. Facetime to reduce behavioral problems in a nursing home resident with Alzheimer's dementia during COVID-19. Psychiatry Research. 2020 **United States**	Dementia, Alzheimer's/ Anxiety	**Aim:** Highlight that interaction with family members using FaceTime improves behavioral problems in patients with Alzheimer's dementia during COVID. The letter addresses that such applications need to be studied both for clinical and research care to be prepared for future pandemics. **Findings:** The patient was reported to communicate well as he could "lip read" during the video session. The daughter reported that her father enjoyed the sessions and seemed to be less anxious and agitated by the end of the video calls. Staff reported that after a series of such video calls, the patient was pacing less and had an improved appetite. The daughter reported that FaceTime had provided a sense of connectedness and had a positive impact on his symptoms.	Case study	N/A
42. Gregucci F et al. Cancer patients in COVID-19 era: swimming against the tide. Radiotherapy and Oncology. 2020, 149, 109-10. **Italy**	Cancer, other /Anxiety	**Aim:** Discuss experiences of working with individuals with cancer and psychosocial considerations. **Findings:** The authors discuss aspects of care such the ''touch" (mandatory for positioning patients) could be experienced as a danger or a consolation for the cancer patient. Most of their patients, during RT, reported feeling of protection by the physician, the radiotherapist, the nurse who wears gloves and masks: ''someone takes care of me concretely". Other patients report fear of being touched, while being carefully positioned on the treatment table. The services described how they decorated the radiotherapy room with Italian landscape to reduce anxiety. Discussed how "the psychological approach for cancer patients is mandatory to	Case example of a radiotherapy service	N/A

Citation and Country	Physical / Mental Health	Aim /Intervention/ Finding(s)	Study Design	Quality[a]
		prevent the fear of infection from causing death by taking the patients away from their life-saving treatments.”		
43. Helmich RC et al. The impact of the COVID-19 pandemic on Parkinson’s disease: hidden sorrows and emerging opportunities. Journal of Parkinson's disease. 2020, 10(2), 351. **USA**	Parkinson’s disease (PD)/ Anxiety	**Aim:** Review the effects of COVID-19 on individuals with PD. **Findings:** Suggests various ways to reduce stress/ examining the determinants of these stress factors (e.g., treatment availability, outlets for stress). The authors indicate that self-management strategies that reduce stress (e.g., mindfulness), increase coping (e.g., cognitive behavioral therapy) or increase physical exercise (e.g., home-based training programs, alone or in groups) may be helpful.	Commentary	N/A
44. Sockalingam S et al. The Impact of Coronavirus Disease 2019 on Bariatric Surgery: Redefining Psychosocial Care. Obesity. 2020, 28(6), 1010-2. **Canada**	Obesity/ Eating disorders, binge eating disorder, depression	**Aim:** Discuss challenges with physical distancing and self-isolation and reassess current psychosocial models to reimagine new models integrating virtual mental health tools/supports for bariatric patients. **Findings:** Approximately 70% of bariatric surgery candidates have a lifetime history of psychiatric illness, which could be exacerbated by COVID-19 pandemic–related stress. Data from SARS suggest that past psychiatric illness is a risk factor for developing active psychiatric illness after the pandemic. Extrapolating from these findings, bariatric patients may be at risk for mental health decompensation, which could impact obesity treatment adherence and long-term patient outcomes. Suggestions for interventions included remotely delivered CBT by telephone or videoconferencing for those with binge eating, emotional eating, depression, and anxiety, patient safety protocols (e.g., remote management of suicidal ideation), virtual care tools, such as app-based tools, and obesity-specific support for social support. The authors identified that although synchronous (e.g., “Zoom groups”) and asynchronous bariatric surgery–specific groups (e.g., Facebook) may be options, issues of privacy and credibility of the information shared should be considered. Mobile and online technologies (e-health) have the	Perspective paper	N/A

Citation and Country	Physical / Mental Health	Aim /Intervention/ Finding(s)	Study Design	Quality[a]
		reported efficacy for psychological symptoms and eating psychopathology.		
45. Minihan E et al. COVID-19, Mental Health and Psychological First Aid. Irish Journal of Psychological Medicine. 2020, 1-2. **Ireland**	Respiratory, asthma, COPD, hypertension, CVD/ Depression, anxiety, anxiety disorders, substance use	**Aim:** Provide an overview of factors elicited in response to COVID-19 and their impact on immunity, physical health, mental health and wellbeing **Findings:** Explores "Psychological First Aid" as a useful technique to help people in this time of crisis. Highlights risk factors for stress and impacts on mental and physical health.	Review	N/A
46. Yan F et al. The role of head and neck cancer advocacy organizations during the COVID-19 pandemic. Head & Neck. 2020, 1–7. **United States**	Cancer, other (head and neck)/ Anxiety, psychosis/psychotic disorder	**Aim:** Outline some of the issues faced by patients with Head and Neck cancer during the current crisis and provide examples of programs being developed by advocacy groups to address them. **Findings:** Discusses advocacy support by professional organizations and HNC advocacy groups and how they responded promptly to concerns of patients by publishing numerous web-based articles, webinars, and podcasts to accurately inform patients of the impact of COVID-19. The groups have also provide online supports and financial assistance e.g., gas cards, collaborations with industry to provide waivers for drug costs, COVID-19-related webinars to help navigate HNC patients through these financially difficult times, assisting low-income HNC patients with the costs of nutritional supplements, medical supplies not covered by insurance, copays, transportation, lodging, and dental care. CancerCare, a general oncology advocacy group, help with offsetting costs of general household expenses, transportation, childcare, and medication.	Review	N/A
47. Fortuna LR et al. Inequity and the disproportionate impact of COVID-19 on communities of color in the United States: The need for a trauma-informed social	Refer to poor physical health / Post-traumatic stress disorders, general stress	**Aim:** Provide insight into the public health impacts of COVID-19 on communities of colour. **Findings:** Guidelines try to address challenges faced by racialized communities dealing with COVID-19 to prevent mental health problems for youth of colour. Highlighted the need for community and cross-sector collaborations and movement away from practices and	Commentary	N/A

Citation and Country	Physical / Mental Health	Aim /Intervention/ Finding(s)	Study Design	Quality[a]
justice response. Psychol Trauma. 2020, 12(5), 443-445. **USA**		regulations that silo clinical and social services, emphasizing respectful, thoughtful, and consistent leadership to empower community stakeholders to address their specific communities' needs; and building capacity around community–academic telehealth partnerships.		
48. Anderson M et al. Developing a sustainable exit strategy for COVID-19: health, economic and public policy implications. Journal of the Royal Society of Medicine. 2020, 113(5), 176-8. **United Kingdom**	Some chronic physical health examples highlighted e.g., Coronary occlusion / Depression (primary), anxiety	**Aim:** Explain the economic perspective and public policy perspective for exit strategies for COVID-19. **Findings:** From an economic perspective, there will be a need to ensure a rapid but sustainable recovery. The International Monetary Fund estimates a 6.1% decline in gross domestic product in advanced economies in 2020,11 although other analyses suggest the decline could be greater. Estimates from Germany suggest that a one-month lockdown would cost between 4.3% and 7.5% of gross domestic product (E150–260 billion). From a public policy perspective, there are several challenges to navigate. First, countries are using technologies in novel ways to ease social distancing measures that may impinge upon civil liberties. For example, China is using an app to monitor people's movement, classifying them according to their health status and travel history.	Commentary	N/A
49. Ajilore O, Thames AD. The Fire This Time: The Stress of Racism, Inflammation and COVID-19, Behavior, and Immunity. 2020, doi: https://doi.org/10.1016/j.bbi.2020.06.003 **USA**	Co-/Multi-morbidities, CVD, lung cancer, type 2 diabetes, hypertension, asthma/ Depression (primary), anxiety, Psychosis/Psychotic disorder	**Aim**: Determine the impact of racial stress on African Americans on inflammatory conditions during the COVID-19 pandemic. **Findings:** Systemic racial discrimination among this population has negative effects on their biological function. First, altered immune function and metabolic changes secondary to stress can contribute to medical comorbidities such as type 2 diabetes, hypertension and asthma, all of which increase COVID-19 risk. Hence, discrimination experiences may also explain why African Americans are at a disproportionately higher risk for poor medical (e.g., cardiovascular disease, metabolic, hypertension) and psychiatric outcomes (e.g., depression, anxiety).	Commentary	N/A

Citation and Country	Physical / Mental Health	Aim /Intervention/ Finding(s)	Study Design	Quality[a]
50. Mediouni M et al. COVID-19: How the quarantine could lead to the depreobesity. Obesity Medicine, 2020, 19, 100255. doi: 10.1016/ j.obmed.2020.100255 **USA**	Obesity / Depression	**Aim:** Examine how the frustration and lifestyle associated with quarantine could lead to 'depreobesity'. **Findings:** Psychologists should play a role in helping individuals deal with stress/ depression during quarantine. Policy makers should begin preparing for the upcoming epidemic of 'deprobesity'. This paper helps highlight potential increases in rates of depression and obesity, which could help policy makers prepare for the period after COVID-19.	Commentary	N/A
51. Shiau S et al. (2020). The Burden of COVID-19 in People Living with HIV: A Syndemic Perspective. AIDS and Behavior. doi:10.1007/s10461-020-02871-9 **USA**	HIV/Psychosocial concerns	**Aim:** Address the burdens faced by PLWH during the COVID-19 pandemic using a syndemic framework, and consequently inform the development of health programming services. **Findings:** The challenges faced by PLWH must be conceptualized using a syndemic framework in order to effectively account for the psychosocial burdens faced by patients.	“Notes from the Field” commentary	N/A
52. Tsamakis K et al. COVID-19 related stress exacerbates common physical and mental pathologies and affects treatment (Review). Experimental and Therapeutic Medicine. doi:10.3892/etm.2020.8671 **Greece**	CVD, myocardial infarction /Anxiety	**Aim:** Discuss the impact of COVID-19 related stress in Cardiology, Paediatrics, Oncology, Dermatology, Neurology, and Mental Health. **Findings:** Maintaining effective communication within the healthcare setting, remaining alert, and introducing novel ways of clinical interactions can help combat the effects of COVID-19 related stress.	Review	N/A
53. Mills JP et al. COVID-19 in older adults: clinical, psychosocial, and public health considerations. JCI insight. 2020, 5(10).	Diabetes (type 2)/ Anxiety, Stress, psychosocial affects of isolation	**Aim:** Discuss the clinical, psychosocial and public health considerations of COVID-19 in the aged population, specifically those with medical comorbidities. **Findings:** The article presents the clinical and psychosocial considerations. In particular, innovative models such as using nursing facilities for short-stay	Viewpoint	N/A

Citation and Country	Physical / Mental Health	Aim /Intervention/ Finding(s)	Study Design	Quality[a]
**USA**		rehabilitation, long-term acute care hospitals for patients requiring long-term ventilation, house calls, and hospital-at-home programs to allow seniors to "age-in-place," have moved health care out of the hospital walls, and helped older adults recover from acute illness and maintain their functional independence.		
54. Nguyen NP et al. Older cancer patients during the COVID-19 epidemic: Practice proposal of the international geriatric radiotherapy group. Cancers. 2020, 12(5), 1287. **USA, France, Switzerland, Spain, Italy, Romania, United Kingdom, Canada, Malaysia, Portugal**	Cancer/ Anxiety, Depression	**Aim:** Highlight the special considerations required to prevent infection of older cancer patients and to provide them with adequate social support during their cancer treatment in the time of the pandemic. **Findings:** Recommendations made: staff education for clinicians (e.g., pathogen infectivity, how to avoid infection when working with patients); a need for personalized care for patients (based on physical performance and SES); scheduling patients with consideration of their transportation availability to minimize interactions; a frailty assessment for older adult cancer patients who require radiotherapy and subsequent hypofractionation when appropriate to reduce total treatment time; social services such as patient navigators should be provided to ensure adequate medical supply, food, and daily transportation to cancer centers, telemedicine for follow-up as well as for those with depression (can be exacerbated during quarantine) and those infected with COVID-19.	Expert Review	N/A
55. Porcelli P. Fear, anxiety, and health-related consequences after the COVID-19 epidemic. Clinical Neuropsychiatry: Journal of Treatment Evaluation. 2020, 17(2), 103-11. **Italy**	Co- and multi-morbidities: CVD, obesity, hypertension, diabetes, stroke, cancer, asthma, COPD, osteoarthritis/	**Aim:** Discuss fear (in light of the COVID-19 pandemic) and its possible forthcoming consequences on health. **Findings:** Hopelessness, helplessness, and loneliness could maybe become the 'normal' distressing and negative feeling experience of both caregivers and patients - as a result of a lack of interpersonal warmth now apparent in healthcare settings due to COVID-19; Loneliness and experiences of social isolation may have a detrimental effect also at the epigenetic level by shortening the telomere length and thus enhancing biological aging; Psychological distress may have dramatic consequences also on	Perspective article	N/A

Citation and Country	Physical / Mental Health	Aim /Intervention/ Finding(s)	Study Design	Quality[a]
	Anxiety, substance use disorder	lethal effects and mortality; One of the most important underlying mechanisms connecting stress and health negative effects is the strict connection between psychological factors and the immune system.		
56. Mattioli AV et al. COVID-19 pandemic: the effects of quarantine on cardiovascular risk. European Journal of Clinical Nutrition. 2020, 1-4. **Italy**	CVD / Anxiety	**Aim:** Discuss the impact of quarantine on cardiovascular risk. **Findings:** Reduced access to food, ability to exercise and engage socially may impact the nutritional and physical activity habits of quarantined persons, resulting in lifestyle changes that are linked with increased cardiovascular disease and obesity. The essay proposed the following interactions: **diet:** decreased access to fresh foods and limited ability to purchase food may result in poor nutritional habits (i.e. increased reliance on processed, limited consumption of fresh fruits and vegetables) and poor eating behaviours caused by anxiety related to the pandemic (i.e. stress-eating).	Perspective	N/A
57. Razai MS et al. Mitigating the psychological effects of social isolation during the COVID-19 pandemic. BMJ. 2020, 369. **United Kingdom**	COPD / Anxiety	**Aim:** Describe how social prescribing can improve the social and psychological wellbeing of patients by drawing from community resources such as the arts (for example, singing in a virtual choir, dancing, or online drawing classes). It can be delivered by trained non-clinical staff in primary care. **Findings:** Social prescribing can improve the social and psychological wellbeing of patients by drawing from community resources such as the arts (for example, singing in a virtual choir, dancing, or online drawing classes). It can be delivered by trained non-clinical staff in primary care	Practice pointer	N/A
58. Balachandar V et al. Follow-up studies in COVID-19 recovered patients-is it mandatory?. Science of The Total Environment. 2020, 139021. **India**	CVD, myocardial infarction, Respiratory, Parkinson's disease, COPD, acute kidney injury/	**Aim:** Review the possible outcomes of COVID-19 recovered patients and determine if they have any other detrimental illnesses (i.e., various multi-organ damages). **Findings:** Brain: site of symptoms is the olfactory bulb, leading to smell dysfunction, Eye: SARS-CoV-2 may enter through the tears and transmit to other organs, Lung: damages the walls and lining cells, causing inflammation, Heart: inflammation can lead to myocardial	Review	N/A

Citation and Country	Physical / Mental Health	Aim /Intervention/ Finding(s)	Study Design	Quality[a]
	Depression, Anxiety	infarction, GI system: early GI symptoms and possible liver damage, Renal: multi-organ damage, Mental health aspect: psychological symptoms such as depression and anxiety may result in addition to these PH conditions after infection<br>Rapid follow-up should be offered to COVID-19 recovered patients, including nucleic acid tests; family members should be trained in empathy skills; the community/society should be educated not to stigmatize recovered patients		
59. Da BL et al. COVID-19 hangover: a rising tide of alcohol use disorder and alcohol-associated liver disease. Hepatology. 2020 **USA**	Co- and multimorbidities, Metabolic syndrome, chronic kidney disease, obesity, alcoholic hepatitis/ Substance use disorder	**Aim:** Examine the vulnerability of patients with Alcohol Use Disorder (AUD) and Alcohol-associated Liver Disease as they have a depressed immune system. Not only are they at risk for COVID-19 but it can further damage the liver as well as social isolation can lead to increased drinking. **Findings:** Suggests the use of telehealth for alcohol counseling and addiction treatment. Integration of AUD/ALD clinics post-COVID-19. Suggests the use of telehealth for addiction medicine as well as improving addiction medicine measures post COVID-19.	Commentary	--
60. Khan S et al. Impact of coronavirus outbreak on psychological health. Journal of Global Health. 2020, 10(1). **China**	Cardiovascular/ Anxiety, depression, PTSD, panic attacks, sleep disturbances	**Aim:** Assess the various effects of COVID-19 on psychological health and suggests interventions that may prove to be helpful. **Findings:** There are numerous interventions suggested by the paper. These include psychological counseling-based therapies (tele-medicine, counseling sessions, cognitive behaviour therapy) and pharmacological treatments.	Commentary	--

[a]Scores given are based on the Mixed Methods Appraisal Tool (#yes/5). No scores assigned to narrative reviews, opinion papers, commentaries, or case studies

Table S3: Chinese Literature Data Extraction Tables (n=17)

Citation	Physical / Mental health	Aim /Intervention/ Finding(s)	Study Design	Study quality[a]
1. Zhang Cang et al. Analysis of the psychological status of diabetic patients during the epidemic of novel coronavirus. Chinese General Practice Nursing. 2020, 18(8), 965-968.	Type 2 diabetes / Anxiety, depression	**Aim:** Investigate the status of mental health of people with diabetes during this pandemic. **Findings:** Approximately 51% of COVID-19 patients have pre-existing chronic conditions and diabetes comprised 20% of those cases. Living area, whether or not they are diagnosed COVID-19 cases around the participants, whether or not the participants have sufficient face masks and diabetic medication, whether or not the participants have an understanding about the status of the pandemic, whether or not they are worried about the pandemic, or following medical advice impact the level of depression (p<0.05). Being worried about the pandemic and following medical advice will impact the level of anxiety. Living area/place and whether or not there is diagnosed case of COVID-19 will impact the level of depression in those with diabetes (p<0.05).	Quantitative Non-Randomized	100%
2. Zhang Jinlong et al. Anxiety and depression status of elderly patients and its influencing factors during the epidemic of novel coronavirus pneumonia. Chinese Journal of Multiple Organ Diseases in the Elderly. 2020, 19(4), 246-250.	Comorbidities, multi-morbidities/ Anxiety	**Aim:** Understand factors of depression and anxiety among older adults who were in quarantine and to suggest interventions for depression and anxiety. **Findings:** 86 participants had an overall average anxiety rating of 31.25 ~ 82.21 (54. 88 ±11.84). 85 had some level of anxiety (98.84%), out of which there were 20 with mild anxiety (23.53%), 12 with severe anxiety (14. 12%) and 53 with moderate anxiety (62.35%). 86 had an overall depression rating of 26.25 ~ 86.42(52.20 ±13.36). 68 cases had some of level of depression (79.07%) and 18 cases had no depression (20.93%). Of those who were depressed, 44 (64.71%) were mildly depressed, 18 (26.47%) were moderately depressed, and 6 (8.82%) were severely depressed.	Quantitative Non-Randomized	100%
3. Cao Jing et al. A survey of anxiety, depression and influencing factors in patients with novel coronavirus pneumonia. Journal of Nursing Science. 2020, 35(9), 15-17.	Comorbidities, multi-morbidities/ Anxiety, depression	**Aim:** Understand COVID-19 patients' psychological status and the contributing factors. **Findings:** Of 148 patients with COVID-19 recruited, 78.38% and 50.00% had no anxiety and depression, respectively; 20.27% and 1.35% had mild and moderate anxiety, respectively; 31.08%, 17.57%, and 1.35% had mild, moderate, and severe depression, respectively.	Quantitative Non-Randomized	100%

Citation	Physical/ Mental health	Aim /Intervention/ Finding(s)	Study Design	Study quality[a]
4. Zhao Miaomiao et al. Investigation on mental status of patients with hematological malignancies during the COVID-19 outbreak and related influencing factors. Nursing of Integrated Traditional Chinese and Western Medicine. 2020, 6(3), 38-41.	Cancer / Anxiety	**Aim:** Understand psychological status of cancer patients during the pandemic and to analyze the contributing factors. **Findings:** 70% of participants had anxiety and 63.33% had depression. Factors impacting anxiety were gender, marital status, being hospitalized or not, and whether they clearly knew preventive measures. Factors impacting depression included gender, age, household income, and hospitalization status (yes/no) (p<0.05).	Quantitative Non-Randomized	100%
5. Chen Xuehong et al. Psychological distress and influencing factors of cancer patients during the new coronary pneumonia epidemic. Chinese Journal of Health Psychology. 2020 [pre-print]. Accessed: https://kns.cnki.net/kcms/detail/11.5257.R.20200720.1025.002.html	Cancer/ Anxiety	**Aim:** Investigate the status of psychological distress and its influencing factors in individuals with tumors during the pandemic of COVID-19. **Findings:** The study was conducted with 189 participants with tumors from 5 tertiary hospitals in Guangdong province. 50.79% exhibited psychological distress. The more impact that COVID-19 had on surgery and treatment, the higher the distress level. 22.80% could not see a doctor as per usual frequency.	Quantitative Non-Randomized	100%
6. Xu Haiyan et al. Optimization to solve the diagnosis and treatment problems and psychological problems of lung cancer patients during the new coronavirus pneumonia epidemic. Chin J Lung Cancer. 2020, 23(4), 247-254.	Lung cancer/ Anxiety	**Aim:** Investigate medical needs of patients with lung cancer and their mental health status during the epidemic periods, in order to provide rational recommendations for subsequent diagnosis and treatment. **Findings:** Out of 368 questionnaires collected, most had confidence in defeating COVID-19, however, 40-75% struggled with nervousness, anxiety, sleep disorders, anger, and sadness. 80% were satisfied with 1-2 online consultation(s).	Quantitative Non-Randomized	80%

Citation	Physical / Mental health	Aim /Intervention/ Finding(s)	Study Design	Study quality[a]
7. Zhou Lixiang et al. Investigation and Analysis of Digestive Endoscopy Patients. Anxiety in Diagnosis and Treatment during the Novel Coronavirus Pneumonia Epidemic. General Nursing. 2020, 18(11), 1349-1351.	Digestive endoscopy patients/ Anxiety	**Aim:** To analyze the digestive endoscopy patients' anxiety level and treatment options during the pandemic. **Findings:** Educational level affected State-Anxiety Index (S-AI) results (P<0.05). S-AI and Trait-Anxiety Index results increased when patients know someone who has been diagnosed with COVID-19. 76.27% willingly pushed back the date of their endoscopy check-up. 80.50% were worried about the delayed check-up's effect on their digestive disorders. When family accompanied the individual, decreased anxiety levels are experienced.	Quantitative Non-Randomized	80%
8. Han Zihui et al. On the psychological intervention of patients with novel coronavirus pneumonia (COVID-19). Medical Dietetics and Health. 2020, Issue 6	Respiratory condition/ Anxiety	**Aim:** Analyze the mental health of COVID-19 patients and to provide intervention guidelines. **Findings:** Self-Rating Anxiety Scale (SAS) scores decreased after mental health interventions. On the second day of admission, the SAS was assessed: 10 cases (13.88%)- below cut-off (<50 points), 33 cases (45.83%) - mild anxiety (50-59 points), 20 cases (27.77%) - moderate anxiety (60-69 points), and 9 cases (12.50%) - severe anxiety (≥70 points). On the fifth day of admission, the SAS scale was re-assessed after the symptomatic psychological intervention was introduced. Among them, 30 (41.66%) were below the cut-off (<50 points), 25 (34.72%) had mild anxiety (50-59 points), 13 (18.05%) had moderate anxiety (60-69 points), and 4 (5.55%) had severe anxiety (≥ 70 points).	Quantitative Non-Randomized	100%
9. Huang Binying et al. Investigation and Prevention and Control Analysis of Elderly Mental State in the New Coronavirus Pneumonia Epidemic. General Nursing. 2020, 18(8), 935-937.	Comorbid chronic disease, old age, frail or frailty/Anxiety	**Aim:** Investigate the mental health (anxiety) of seniors during the pandemic, provide interventions and suggest next steps to protect their mental health. **Findings:** 70% of participants had anxiety and 63.33% of participants had depression. Factors impacting anxiety included gender, marital status, hospitalized or not, whether they clearly know preventive measures. Factors impacting depression included gender, age, household income, and hospitalization status (yes/no) (p<0.05)	Quantitative Non-Randomized	100%

Citation	Physical / Mental health	Aim /Intervention/ Finding(s)	Study Design	Study quality[a]
10. Mu Chanjuan. Survey on the awareness and psychological status of new coronavirus pneumonia among middle-aged and elderly residents. Chinese General Practice Nursing. 2020, 18(8), 952-955.	Comor-bidities, multi-morbidities/ Anxiety, depression	**Aim:** Investigate community dwelling middle and older adults' knowledge related to COVID-19, awareness of COVID-19, and psychological status. **Findings:** Phone interviews were conducted with 217 individuals. The results showed that 23 people had depression or anxiety. Only 11 people had anxiety, 6 people had depression and 6 people had both depression and anxiety. Age and the presence of comorbidities/multi-morbidities were factors associated with anxiety. The presence of comorbidities/multi-morbidities was the factor associated with depression.	Quantitative Non-Randomized	100%
11. Wang Mengyu et al. Mental health status of middle-aged and elderly people under the new coronavirus pneumonia epidemic. Chin J Mult Organ Dis Elderly. 2020, 19(4), 241-245.	Old age/ Anxiety, depression	**Aim:** Investigate the psychological status of middle and older adults during the pandemic. **Findings:** 8.5% had moderate to severe depression and 10.7% had moderate to severe anxiety. 50.7%, 30.8%, 11.7%, and 6.8% had no, mild, moderate, and severe depression, respectively. 63.1%, 26.2%, 7.9%, 2.8% had no, mild, moderate, and severe anxiety, respectively.	Quantitative Non-Randomized	80%
12. Wu Lili et al. Investigation of Post-traumatic Stress Symptoms in 2 Confirmed and 6 Suspected New Coronavirus Pneumonia Patients. Journal of Second Military Medical University 2020, 41(2), 186-190	Respiratory, other/ Post-traumatic stress disorder	**Aim:** Explore the psychological status and interventions in confirmed and suspected cases of COVID-19. **Findings:** The data of 2 confirmed cases and 6 suspected cases of COVID-19 were collected through an online survey. Demographic data were collected by a self-designed self-assessment general situation questionnaire. Post-traumatic stress symptoms were assessed by post-traumatic stress disorder checklist of the DSM-5 (PCL-5). One person screened positive for PTSD; there was one suspected case. Invasive symptoms presented in 5 cases.	Quantitative Non-Randomized	60%

Citation	Physical / Mental health	Aim /Intervention/ Finding(s)	Study Design	Study quality[a]
13. Zhang Yuping et al. Psychological stress and protective countermeasures of tumor patients during the epidemic period of new coronavirus pneumonia. Tumor Prevention and Treatment，2020，33(2), 106 - 110.	Cancer/ Anxiety, depression	**Aim:** Lay a foundation for strengthening crisis management and improving strategies on intervention and management in psychological stress of cancer patients during the outbreak of the epidemic: **Professional intervention:** [1] Cognitive behavioural therapy (CBT); [2] mindfulness-based stress reduction (MBSR); [3] Narrative therapy. The 7-step prevention method included: Self-regulation includes accepting own's emotions, building trust with health care professionals, regular daily routine, connect with friends and family, entertainment and physical activity, preparing for a good night's sleep, and contacting doctors if symptoms worsen	Qualitative	60%
14. Zhou Jin et al. Management and practice of mental health of hemodialysis patients during the novel coronavirus pneumonia epidemic. Nursing of Integrated Traditional Chinese and Western Medicine. 2020, 6(3), pp. 139-141.	Kidney disease/ Mental health	**Aim:** Summarize mental health problems faced by patients undergoing dialysis and the principle of "five in one" as psychological intervention. **Findings:** To ensure there is no interruption on dialysis and that the patients' psychological status is in good state, the department established the principle, "five in one". The principle consists of educating patients about COVID-19, sending messages about mental health, providing psychological support by family and friends, and providing mental health services.	Qualitative	20%
15. Wang Lin et al. Discussion on home care practice for the elderly under the new coronavirus pneumonia epidemic. Journal of Jinan University (Natural Science and Medicine Edition). 2020, 41(2), 123-128.	Frailty/Anxiety	**Aim:** Explore the effect of home care in the elderly under the epidemic situation of COVID-19. **Findings:** Care procedure: [1] Before at-home care: call to evaluate the patient family's contact history with COVID-19; [2] Teach the patient and family members to properly seal masks while maintaining 1~1.5 metres of safe distance; [3] Provide COVID-19 related training to nurses who specialize in home care; [4] Afterwards, evaluate the ability of family members to care for the elderly patient; [5] Educate and provide training on caring for the elderly patient during the pandemic	Quantitative Randomized	60%

Citation	Physical / Mental health	Aim /Intervention/ Finding(s)	Study Design	Study quality[a]
16. Yuan Jinhui and Li Qingsong. Treatment process and doctor-patient protection measures for tumor radiotherapy patients under the epidemic of new coronavirus infection pneumonia. Modern Oncology. 2020, 28(8), 1407-1409.	Cancer/ Anxiety, depression	**Aim:** Provide advice regarding strategies to ensure tumor patient's safety during their medical treatment. **Findings:** The hospital provided an online portal for patients and healthcare workers to ask and answer questions regarding mental health counselling, COVID-19 prevention and new information about the state of COVID-19. This will reduce fearfulness and increase optimistic toward medical treatment and daily life.	Qualitative	80%
17. Chen Qiong et al. Key points for prevention and treatment of new coronavirus pneumonia in the elderly (for trial implementation). Chinese Journal of Geriatrics. 2020, 39(2), 113-118.	Older adults/ Anxiety, depression	**Aim:** Draft key points for the prevention and treatment of the Novel Coronavirus Pneumonia in the elderly, aiming to provide help with the prevention and treatment of NCP and the reduction of harm to the elderly population. **Findings:** As an individual ages, the baseline levels of pro-inflammatory cytokines in the tissues and circulation of the elderly increase with age, especially interleukin (IL)-1β, IL-6 and tumor necrosis factor-α (TNF-α), which is a phenomenon is called "Inflammatory aging". while performing clinical classifications, physicians must consider the functional status of other body systems, because older individuals with lung disease may be more likely to induce failure of other systems.	Qualitative	60%

[a]Scores derived from the Mixed Methods Appraisal Tool (#yes/5)
